# The Development and Content of Movement Quality Assessments in Athletic Populations: A Systematic Review and Multilevel Meta-Analysis

**DOI:** 10.1186/s40798-025-00813-0

**Published:** 2025-01-23

**Authors:** Gyan A. Wijekulasuriya, Carl T. Woods, Aden Kittel, Paul Larkin

**Affiliations:** 1 Institute for Health and Sport, Victoria University, Melbourne, Australia; 2 MSA Research Centre, Maribyrnong Sports Academy, Melbourne, Australia; 3 School of Human Movement and Nutrition Sciences, The University of Queensland, St Lucia, Australia; 4 Centre for Sport Research, School of Exercise and Nutrition Sciences, Deakin University, Melbourne, Australia

**Keywords:** High performance sport, Functional movement, Movement screening, Movement competency

## Abstract

**Background:**

Despite their prominence in the sport and human movement sciences, to date, there is no systematic insight about the development and content of movement quality assessments in athletic populations. This is an important gap to address, as it could yield both practical and scientific implications related to the continued screening of movement quality in athletic contexts. Hence, this study aimed to systematically review the (i) *developmental approach*, (ii) *movements included*, (iii) *scoring system utilised*, and (iv) the *reliability* of movement competency assessments used in athletic populations.

**Methods:**

Electronic databases (SPORTDiscus, MEDLINE, CINAHL, Web of Science, Scopus) were searched for relevant articles up to 12 May 2023. Studies were included if they reported data about the *developmental approach*, *movements included*, *scoring system utilised* and *reliability* of assessment in an athletic population. A modified Downs and Black checklist was used to measure study quality.

**Results:**

From a total of 131 identified studies: (i) 26 (20%) described the *developmental approach* of an assessment; (ii) 113 (86%) included descriptions of the *movements included*; (iii) 106 (81%) included a description of *scoring system and criteria*; and (iv) 77 (59%) studies included *reliability* statistics. There were 36 assessments identified within these studies, comprising 59 movements in total. Each assessment scored movement quality through a Likert or binary classification system.

**Conclusion:**

First, the results demonstrate that choosing an appropriate movement quality assessment in an athletic population may be a complex process for practitioners as the development approach, movements included and scoring criteria vary substantially between assessments. Second, academics could use these results to help design new assessments for novel applications that meet rigour and reliability requirements. Third, these results have the potential to foster guidelines of use for the reliable assessment of movement quality in athletic populations.

**Supplementary Information:**

The online version contains supplementary material available at 10.1186/s40798-025-00813-0.

## Key Points

• The developmental approach, movements included and reliability of movement quality assessments vary within the literature. Given this, it is suggested that practitioners and researchers think critically when selecting an assessment that is appropriate for their context.

• The assessment of composite scores appears more reliable than movement specific scores. Moreover, users of various movement quality assessments should be aware that rater experience can affect assessment reliability.

• The relationship between movement quality assessments and their target application may not be universal. Therefore, the development of new assessments is warranted for emerging applications and to overcome issues in current assessments.

**Supplementary Information:**

The online version contains supplementary material available at 10.1186/s40798-025-00813-0.

## Background

Movement quality is a latent physical attribute defined as an individual’s ability to perform a specific task or movement pattern [1, 2]. Poor movement quality is characterised by movements that result from disrupted agonist, antagonist and/or synergistic muscle function during movement [2]. Athletes that participate in competitive sport perform specialised skills which require the synergistic coordination of multiple muscle groups [2]. Thus, poor movement quality may restrict aspects of skill development in various athletic populations [2]. Hence, developing movement quality may be foundational for the development of physical fitness attributes in athletes [3, 4]. To this end, movement quality has been associated with greater physical fitness [5, 6] and is capable of discriminating talent in team sports [7, 8]. The assessment of movement quality, and its ensuing development over time, is thus an important consideration for practitioners and athletes in sporting contexts due to purported beneficial effects related to sport performance.

Movement quality assessments are categorised as ‘process’ assessments [2, 3, 7–9] as they direct attention toward movement performance (i.e., how ‘well’ a movement was performed). Examples include the Athletic Ability Assessment (AAA) [10], Movement Competency Screen [1] and Functional Movement Screen (FMS) [11, 12]. While each assessment has been developed for different purposes, they tend to assess movement using similar components. These components include assessing multiple movements through a standardised scoring system, grounded in pre-determined criteria. Scoring systems are typically aggregated across body segments to produce a score for each movement and movement scores are summated into a composite score. Thus, while scoring is somewhat subjective (i.e., based on a practitioner associating movement relative to a criterion), they can provide information about areas of (dys)function, which may result in targeted exercise prescription [10].

Due to the generality of its definition, a range of movement quality assessments exist across the literature [e.g., 3, 13–15]. Since assessments are developed for various purposes [2], they typically integrate different methods. This means each assessment may use unique movements, scoring systems and criteria for evaluation. Hence, researchers and practitioners are faced with the challenge of choosing an assessment that aligns with their needs [2, 3]. For instance, some assessments are designed for specific sporting populations [16], for identifying movements that may be poorly executed during resistance training [17], to guide specific conditioning activities [18], or to assess whole body movement quality during athletic activities [7, 10, 18]. Summarising the content of these assessments may help researchers and practitioners in this selection process.

Currently, two reviews have described the properties of movement quality assessments relevant for athletic populations. One reported the properties of multicomponent musculoskeletal movement quality assessments [2], and the other the content of movement quality assessments that evaluate athletic motor skills [3]. While of importance for the field, these reviews did not include a wide range of assessments specific to athletic populations due to their respective inclusion criteria and scope of analysis [2, 3]. Moreover, the developmental approach, which encompasses the purpose of designing an assessment and its ensuing methods, is scantly discussed in the literature. A detailed analysis of these features could thus be used to infer an assessment’s intended use and content validity (i.e., how well the assessment measures movement quality) [19]. To date, no study has summarised the developmental approach for all movement quality assessments used in athletic populations. The results of such a review would be a useful resource for those interested in gaining richer insight as to the most appropriate assessment for their context.

A systematic review that focuses on the development and content of movement quality assessments should consider a few key components. The first relates to the *developmental approach* of a movement quality assessment, which implicates how movement quality is operationalised [13]. Therefore, the *purpose of development* and *method of development* of movement quality assessments are important components to understand. The second relates to the *movements included* in an assessment, and their respective body regions. Following this, the third relates to the *scoring systems* and subsequent criteria utilised. A fourth component – the *reliability* of the scoring criteria – could also implicate a fifth component – the *technical error* and *minimal detectable change* of an assessment. Conducting such a review would likely identify popular and reliable assessments of movement quality, while highlighting various developmental approaches. This information may guide researchers and practitioners when making informed decisions regarding the assessment selection. Further, the findings may provide guidance for the development of new assessments by identifying areas of strength and growth in current assessments. Our primary aim, here, was to systematically review the movement quality literature with regards to the (i) *developmental approach*, (ii) *movements included*, and (iii) *scoring systems* utilised for movement quality assessments used within athletic populations. A secondary aim was to conduct a meta-analysis to investigate the: (iv) intra- and inter-rater *reliability* of the identified assessments.

## Methods

### Search Strategy

The search strategy was registered with PROSPERO prior to the initial search (CRD42023425747) and followed PRISMA guidelines (see Online Resource 1). The search strategy was intended to meet the needs of this review, while paving the way for a broader project aiming to identify the content, reliability and association with physical fitness of movement quality assessments in athletic populations. Electronic databases (SPORTDiscus, MEDLINE, CINAHL, Web of Science, Scopus) were searched and articles related to the developmental approach, movements included, scoring methods and their subsequent reliability were identified. Studies included had to be written in English and published in peer-reviewed journals from 1 January 1990 to 12 May 2023. Search phrases were determined by a steering committee of content experts. The search terms and Boolean operators used are presented in Table 1. The reference list of all studies that underwent full-text review were inspected for relevant articles.

**Table 1:** Search terms used in the systematic review

General Term	Search Term
1. Movement Quality	motor competency OR functional movement OR movement competency OR motor control OR foundational movement OR athletic abilities OR athletic movement
2. Assessment	assess* OR screen* OR tool
3. Physical Fitness	physical performance OR physical fitness OR fitness OR athletic performance OR physical capacity
4. Statistical relationship	relationship OR correlation OR association OR related OR predict*
5. Reliability	reliab* OR rater OR intra OR inter OR kappa statistic
Search phrases	1 AND 2 AND 3 AND (4 OR 5)

### Study Selection and Criteria

All articles identified by the search strategy were imported into an online reference management software (Covidence, Melbourne, Australia). Following the removal of duplicates, title and abstract reviews were conducted by two reviewers. The full text of all remaining articles were reviewed by the same two reviewers using the inclusion and exclusion criteria listed in Table 2. When there was disagreement, a third reviewer determined the suitability of a text for inclusion or exclusion.

**Table 2:** Inclusion and exclusion criteria for the systematic review

Inclusion Criteria	Exclusion Criteria
• Assessment developed for trained participants. • Include data specific to a process movement quality assessment • Describe at least one of the following elements of a movement quality assessment a) Developmental approach b) Movements included c) Instructions for administration of assessment d) Scoring system and/or criteria of assessment e) Sensitivity and/or specificity analysis to determine discriminant validity f) Inter-rater and/or intra-rater reliability g) Technical error h) Relationship with physical fitness measure	• Intervention studies • Any study not specifying the target population, or the target population was not trained as per the below definition.

Note: the definition of ‘trained’ was aligned with recommendations in the literature [24]

### Study Quality Assessment

Study quality was determined using a modified Downs and Black [20] checklist. This checklist was modified from the original to be relevant for methodological studies that report reliability statistics as their primary outcomes. As shown in Table 3, this resulted in a score out of 14 for each included study. Unreported variables were classified as a “no” response. Thresholds for study quality of 50% for fair, 70% for good and 90% for excellent were set in accord with recommendations in the literature [21]. Any studies with scores < 50% were considered poor quality.

**Table 3:** Modified Downs and Black [25] checklist used to assess study quality

Category	Criteria
Reporting	1. Is the hypothesis clearly described? Y/N (1) 2. Are the main outcomes to be measured described in the introduction/methods sections? Y/N (2) 3. Are the characteristics of the participants included in the study clearly described? Y/N (3) 4. Are the main findings of the study clearly described? Y/N (6) 5. Has the study provided values of random variability in the data for main outcomes? Y/N (7) 6. Have actual probabilities been reported for the main outcomes except where the probability value is less than 0.001? Y/N (10)
External Validity	7. Were the subjects asked to participate in the study representative of the entire population from which they were recruited? (11)
Internal Validity (Bias)	8. Was an attempt made to blind participants to the outcomes of the study where relevant? (14) 9. Was an attempt made to blind those assessing to the main outcomes of the study where relevant? (11) 10. Were any of the results a result of p-hacking/data-dredging? (16) 11. Were the statistical tests used to assess the main outcomes appropriate? (18) 12. Were the outcome measures used accurate (valid and reliable)? (20)
Internal Validity (Selection Bias)	13. Was there adequate adjustment for confounding in the analyses from which the main findings were drawn? (25)
Power	14. Did the study have sufficient power to show reliability and/or validity? Was there a power calculation? (27)

Method of assessment is included after the question and the number in brackets refers to the question number in the original Downs and Black checklist. Y/N = yes or no

### Data Extraction

Data were extracted by the first author and collated in a customised Excel spreadsheet, with participant demographic information recorded (age, mass, height, sample size, sport, training history). The following data were extracted for review: *movement quality assessment name*; *assessment developmental approach*; *movements included in the assessment*; *scoring system and criteria*; *reliability*; *technical error statistics;* and *specificity and/or sensitivity analysis*.

### Data Analysis

All descriptive statistics are reported as mean ± standard deviation or percentages. A multilevel meta-analysis was used to summarise reported intraclass correlation coefficients (ICC) of movement quality assessment composite scores [22]. This method was selected to minimise the effect of dependence on the meta-analysis. The ICC values were transformed into Fisher’s z-scores for analysis to redistribute the *r* coefficients to reflect a normal distribution [23]. The results of each meta-analysis were then reverse-transformed into *r* correlation coefficients. The inter- and intra-rater reliability for individual movement scores were summarised using mean Kappa and percentage agreement values. The guidelines of Mukaka [24] were used to interpret magnitude of ICCs, with 0.00 < *r* ≤ 0.30 being negligible, 0.30 < *r* ≤ 0.50 low, 0.50 < *r* ≤ 0.70 moderate, 0.70 < *r* ≤ 0.90 high and *r* > 0.90 for very high associations between variables. Guidelines developed by Landis and Koch [25] were used for the interpretation of summarised Kappa statistics (< 0.20 = slight agreement, 0.21–0.40 = fair agreement, 0.41–0.60 = moderate agreement, 0.61–0.80 = substantial agreement, > 0.81 = almost perfect agreement).

## Results

### Overview of Studies

**Fig. 1:**
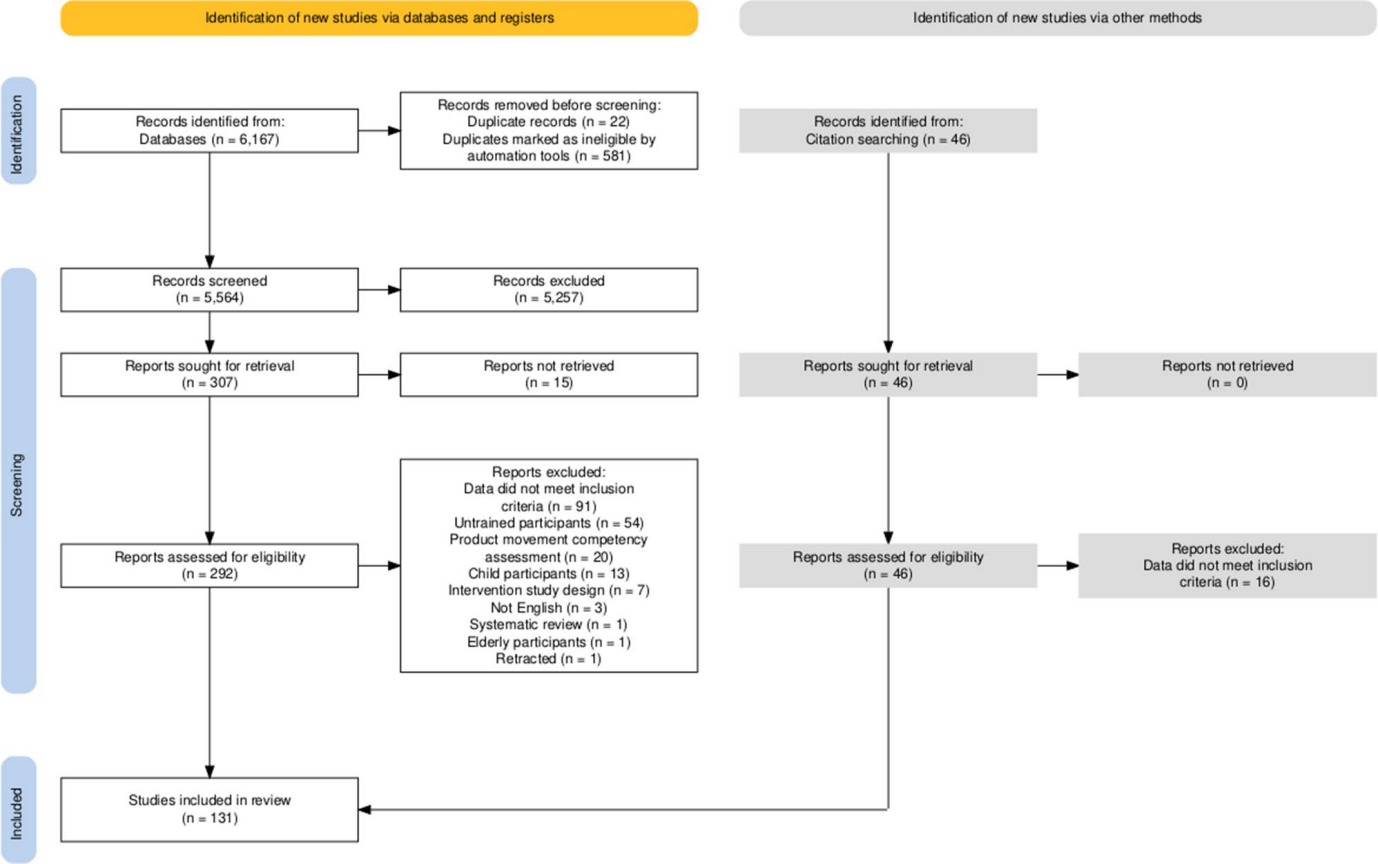
PRISMA study inclusion flowchart

The initial search identified 6,167 studies. After duplicates were removed, 5,564 studies were screened for relevance, 5,257 studies were excluded, and a further 191 were excluded by the inclusion/exclusion criteria during full text review (Fig. 1). An additional 30 studies were identified through the searching of reference lists that met the inclusion criteria, leading to a total of 131 included studies.

Of the 131 studies included (Table 4), 26 (20%) described the *developmental approach* of an assessment, 113 (86%) had descriptions of the *movements included*, 106 (81%) contained a description of *scoring system and criteria* and 77 (59%) studies had *reliability* statistics. The *technical error* and/or *minimal detectable change* (*N* = 10; 7%) and sensitivity and specificity analysis (*N* = 5; 4%) were least reported.

**Table 4:** Characteristics of the study type, population characteristics, movement quality assessment and data included in each study

Reference	Movement Quality Assessment	Data included in study			
		Developmental Approach	Movements	Scoring	Reliability
Alkhathami et al. [26]	FMS		X	X	X
Armstrong et al. [27]	FMS		X	X	X
Armstrong and Greig [28]	FMS		X	X	X
Armstrong [29]	FMS		X		X
Atalay et al. [30]	FMS		X	X	
Bakalar et al. [31]	FMS		X	X	
Bakken et al. [32]	9 + Screening Battery			X	X
Barnett et al. [33]	RTSB		X	X	X
Bennett et al. [34]	FMS				
Bennell et al. [35]	Weight bearing dorsiflexion		X	X	X
Borms and Cools [36]	YBT-UQ, CKCUEST		X	X	X
Bullock et al. [37]	FMS, YBT-UQ		X	X	
Butowicz et al. [38]	Movement system screening tool	X	X	X	X
Butler et al. [39]	FMS-100	X	X	X	X
Campa et al. [40]	FMS		X		
Chang et al. [41]	FMS		X	X	X
Chapman et al. [42]	FMS		X	X	X
Chimera et al. [43]	FMS		X	X	
Clifton et al. [44]	FMS		X		
Conkin et al. [45]	FMS		X		X
Cook et al. [11]	FMS	X	X	X	
Cook et al. [12]	FMS	X	X	X	
Davis et al. [46]	FMS		X	X	
Degot et al. [47]	Modified CKCUEST	X	X	X	X
de Oliveira et al. [48]	FMS		X	X	
Dobbs et al. [89]	Back Squat Assessment		X	X	X
Domaradzki and Kozlenia [50]	FMS		X	X	
Edis [51]	Modified FMS		X	X	
Ferreira et al. [52]	CKCUEST		X	X	
Fox et al. [53]	FMS		X	X	
Frohm et al. [54]	9 + screening battery	X	X	X	X
Frost et al. [55]	FMS		X	X	
Frost et al. [56]	FMS		X	X	
Garrett et al. [7]	AAA		X	X	
Glass et al. [57]	FMS		X		
Glaws et al. [58]	Selective Functional Movement Assessment		X	X	X
Gnacinski et al. [59]	FMS		X	X	X
Goldbeck and Davies [60]	CKCUEST	X	X	X	X
Gonzalo-Skok et al. [61]	Weight bearing dorsiflexion and Modified Star Excursion Balance Test		X	X	X
Gorman et al. [62]	YBT-UQ	X	X	X	X
Gribble et al. [63]	FMS		X	X	X
Harshbarger et al. [64]	FMS		X		
Hartigan et al. [65]	In Line Lunge		X	X	
Hernandez-Garcia et al. [66]	Basic Fundamental Movement Assessment	X	X	X	X
Hollstadt et al. [67]	Modified CKCUEST		X	X	X
Inovero et al. [68]	Movement Competency Screen-2	X	X		X
Ireton et al. [6]	Modified AAA		X	X	X
Jaffri et al. [69]	Dynamic Leap and Balance Test	X	X	X	X
Kara [70]	FMS		X	X	
Kara et al. [71]	FMS		X	X	
Kazman et al. [72]	FMS		X		
Kelleher et al. [73]	FMS				X
Kenji et al. [74]	FMS		X	X	X
Koehle et al. [75]	FMS				X
Kozlenia et al. [76]	FMS		X	X	X
Kozlenia and Domaradzki [77]	FMS		X	X	
Kramer et al. [78]	FMS		X	X	X
Kraus et al. [79]	FMS		X	X	
Krysak et al. [80]	FMS		X	X	
Lee and Kim [81]	CKCUEST		X	X	
Lee et al. [82]	FMS		X	X	X
Lee et al. [83]	FMS		X	X	
Leeder et al. [84]	FMS		X	X	X
Li et al. [85]	FMS				X
Liang et al. [86]	FMS		X	X	
Lisman et al. [87]	FMS		X	X	
Lloyd et al. [88]	FMS		X	X	
Lockie et al. [89]	Modified FMS		X	X	
Lockie et al. [90]	FMS		X	X	
Lockie et al. [91]	FMS		X	X	
Loudon et al. [92]	FMS		X	X	X
Lubans et al. [17]	RTSB	X	X	X	X
Magyari et al. [93]	FMS		X	X	
Mann et al. [94]	Untitled Movement Screen		X	X	X
Matsel et al. [95]	Arm Care Screening Tool	X	X	X	X
McCann et al. [96]	FMS		X	X	X
McKeown et al. [10]	AAA	X	X	X	X
Milbank et al. [97]	Movement Competency Screen		X	X	X
Miller and Susa [98]	FMS		X	X	X
Minick et al. [99]	FMS		X	X	X
Misegades et al. [100]	FMS		X		
Mu et al. [101]	FMS			X	
Myer et al. [102]	Tuck Jump Assessment	X	X	X	X
Myer et al. [103]	Back Squat Assessment	X	X	X	
Okada et al. [104]	FMS		X	X	
Onate et al. [105]	FMS		X	X	X
Padua et al. [106]	LESS	X	X	X	X
Padua et al. [107]	LESS – Real Time	X	X	X	X
Parchmann and McBride [108]	FMS				
Parenteau et al. [109]	FMS		X	X	X
Parsonage et al. [18]	Conditioning-Specific Movement Tasks	X	X	X	X
Pichardo et al. [110]	RTSB		X		X
Popchak et al. [111]	CKCUEST		X	X	X
Pullen et al. [112]	AIMS and Tuck Jump Assessment		X	X	X
Rafnsson et al. [113]	9 + screening battery		X		X
Reid et al. [16]	Netball Movement Screening Tool	X	X	X	X
Rogers et al. [114]	Modified AAA		X	X	X
Rogers et al. [115]	AIMS	X	X	X	X
Rogers et al. [116]	AAA-6	X	X	X	X
Roush et al. [117]	Step Down Test		X	X	
Rowell and Relph [118]	LESS		X		X
Rowan et al. [119]	FMS		X	X	
Schneiders et al. [120]	FMS			X	X
Schwiertz et al. [121]	YBT-UQ		X	X	X
Shaffer et al. [122]	YBT-LQ		X	X	X
Shojaedin et al. [123]	FMS				X
Shultz et al. [124]	FMS			X	X
Sikora and Linuk [125]	FMS				X
Silva et al. [126]	FMS		X	X	
Silva et al. [127]	FMS		X	X	
Silva and Clemente [128]	FMS			X	
Silva et al. [129]	CKCUEST		X	X	X
Smith et al. [130]	FMS		X		X
Smith et al. [131]	FMS		X	X	X
Smith et al. [132]	YBT-LQ				X
Sommerfield et al. [133]	Back Squat Assessment				X
Stepinski et al. [134]	FMS			X	
Terry et al. [135]	Modified Musculoskeletal Readiness Tool	X	X		
Teyhen et al. [136]	FMS		X	X	X
Venter et al. [137]	FMS		X	X	X
Vidal et al. [138]	Overhead Squat	X		X	
Waldron et al. [139]	FMS		X		X
Warshaw et al. [140]	Movement Competency Screen		X		
Whatman et al. [141]	Lower Extremity Functional Tests		X	X	X
Whiteside et al. [142]	FMS		X	X	X
Willigenburg and Hewett [143]	FMS			X	
Woods et al. [5]	Modified AAA	X	X	X	X
Woods et al. [8]	Modified AAA	X	X	X	X
Zalai et al. [144]	FMS		X	X	
Zhang et al. [145]	FMS			X	
Zou et al. [146]	FMS		X	X	

AAA = Athlete Ability Assessment, AIMS = Athlete Introductory Movement Screen, CKCUEST = Closed Kinetic Chain Upper Extremity Stability Test, FMS = Functional Movement Screen, LESS = Landing Error Scoring System, LQ = Lower Quarter, N/A = not applicable, RTSB = Resistance Training Skills Battery, UQ = Upper Quarter, YBT = Y Balance Test

### Assessment of Study Quality

No study fulfilled all criteria in the modified Downs and Black checklist. The highest score was 14/15 and studies were generally of good quality (median study quality score of 67%; see Online Resource 2).

### Developmental Approach

#### Purpose of Development

Of the 26 studies that described the developmental approach of an assessment, 12 stated their purpose was to create an assessment of movement quality that identified movement dysfunction that may be related to greater injury risk, and 11 stated their purpose was to assess movement quality related to physical fitness or athletic motor skill competency (Table 5). One assessment was developed to assess movement quality of fundamental movement skills and one assessment did not state its purpose.

**Table 5:** Developmental approach of the authors of the included movement quality assessments

Study	Assessment	Purpose Category	Method Category	Detailed purpose of development	Detailed method of development
Butowicz et al. [38]	Movement system screening tool	Injury risk	Modified Delphi	The movement system screening tool was designed to assess movement pattern efficiency, regional stability, mobility, and movement symmetry across the core and upper and lower extremities. The authors wished to develop a comprehensive, whole body screening tool with injury predictive validity based on injury risk factors that have been identified in peer-reviewed literature.	A systematic review generated a list of movement quality assessments that were associated with injury in the literature or were commonly used to assess movement patterns, mobility, control of dynamic movements or muscle capacity (strength, endurance). Then, in a modified Delphi approach, an expert panel of physical therapists, athletic trainers, certified strength and conditioning specialists and biomechanists (*n* = 15, experience = 15.1 ± 0.9 y) were surveyed three times. *The questions in round 1 established whether experts agreed that the movements listed assessed either movement pattern efficiency*,* stability*,* mobility or movement symmetry. Experts were also asked if the proposed movements assessed every area of the body.* *In round 2 experts were asked the risk factor assessed by a movement*,* the primary body region and the importance of symmetry during each movement.* *In round 3 experts were provided reliability information for each test and rank-ordered them from most important to assess to least important.*
Butler et al. [39]	FMS-100	Injury risk	Re-designed (Sensitivity)	The FMS-100 was developed to increase the sensitivity of the FMS [15, 16] in response to motor control interventions and to detect injury risk.	In the FMS [15, 16], each movement is scored equally (/3) but the authors re-designed the FMS by re-weighting the scoring of movements (8–20 points) with higher scores allocated to movements that require greater neurodevelopment (e.g. deep squat).
Cook et al. [11], Cook et al. 12]	Functional Movement Screen	Injury risk	Self-selected	The FMS was designed as a pre-participation screen that attempts to assess the ability of an individual to execute fundamental movement patterns. Moreover, the authors suggested that the results of the screen can be used to individualise strength and conditioning programs to improve function which may reduce injury risk and increase physical fitness.	The authors developed an assessment of the mobility and stability of the whole body using self-selected movements and criteria. These criteria were informed by proprioceptive and kinaesthetic awareness principles that state that dysfunction proximal to the body will affect function in distal body segments. Hence, dysfunction driven by poor control of segments proximal to the torso scores poorly (1, 2) and a score of 3 indicates no functional impairments for a movement.
Degot et al. [47]	Modified Closed Kinetic Chain Upper Extremity Stability Test	Injury risk	Re-designed	The CKCUEST [56] was modified to improve its validity by normalising the distance between each hand to an individual’s arm span.	The authors modified the CKCUEST by normalising the distance between each hand to an individual’s arm span. The authors suggested this would reduce the variation in scapular position and shoulder activation between individuals and improve consistency in the test’s application in the field.
Frohm et al. [54]	9 + screening battery	Physical fitness	Self-selected	The 9 + screening battery was developed to be a functional movement screen that can be used to assess movement quality in athletes.	The authors reported the movements and assessment criteria which were developed and used in the assessment processes of a national sporting organisation. These movements included: 1) 6 movements from the FMS with redeveloped assessment criteria 2) the one-legged squat from the United States Tennis Association High Performance Profile 3) the straight leg raise and seated rotation test developed by the authors of the study.
Goldbeck and Davies [60]	Closed Kinetic Chain Upper Extremity Stability Test	Injury risk	Self-selected	The authors developed the closed kinetic chain upper extremity stability test to determine whether there are deficits in closed kinetic chain upper extremity functional performance and to guide rehabilitation.	The lead author piloted the test for several years prior to publishing its procedures.
Gorman et al. [62]	Y Balance Test – Upper Quarter	Injury risk	Self-selected	The authors developed the assessment to overcome the limits of previous assessment of upper body function. This assessment requires thoracic and scapular mobility and dynamic stability in contrast to other assessments of upper body function [55, 56].	The authors developed the test to determine mobility and stability of the upper body using the Y Balance Test kit.
Hernandez-Garcia et al. [66]	Basic Fundamental Movement Assessment	Fundamental movements	Modified Delphi	This assessment was developed to be an easy, simple and concise protocol to summarise the quality of fundamental movement patterns.	The authors selected five movements from the movement competency assessment literature then expert judges (*n* = 10, no description of profession) determined *the compensations that indicate non-functional execution that is related to injury during each movement*.
Inovero et al. [68]	Movement Competency Screen − 2	Physical fitness	Re-designed	The authors added 5 movements, with greater complexity, to the Movement Competency Screen [1].	The authors selected 5 new movements (bilateral countermovement jump, bilateral countermovement jump to a unilateral land, bilateral broad jump to a unilateral land, explosive push up, bend-and-pull at speed) to add to the Movement Competency Screen [1].
Jaffri et al. [69]	Dynamic Leap and Balance Test	Physical fitness	Self-selected	The authors wished to create an assessment that measured dynamic balance abilities during a functional jumping task in which an athlete alternates the weight bearing limb.	The authors adapted scoring systems and criteria in the Balance Error Scoring System and Y Balance Test – Lower Quarter and used them to assess a leap and balance task.
Lubans et al. [17]	Resistance Training Skills Battery	Physical fitness	Modified Delphi	The Resistance Training Skills Battery was developed for the following purposes: 1. To evaluate the efficacy of school- and community- based resistance training programs 2. To assess individual progress and provide feedback in resistance training programs 3. To use in research as a measure of movement skill competency in adolescent populations.	An initial assessment was developed by the authors who the asked for feedback from a group of 14 experts in youth resistance training (PhD qualified and published in the area). Eight experts responded and provided feedback on: 1) *The importance of developing a resistance training skills battery* 2) *The selected exercises* 3) *The assessment criteria for each exercise* These responses were used to inform the design of the final battery.
Mann et al. [94]	Untitled Movement Screen	N/A	Self-selected	There was no reported purpose for developing this screening tool.	The authors self-selected movements and assessment criteria from previous movement screens that were “sport-specific”.
Matsel et al. [95]	Arm Care Screening Tool	Injury risk	Self-selected	The Arm Care Screening Tool was designed to assist high school baseball coaches screening for movement dysfunction that may increase the risk of injury during baseball specific movements in adolescent athletes.	The authors selected movements from other screens that were relevant for baseball.
McKeown et al. [10]	Athlete Ability Assessment	Physical fitness	Self-selected	The Athlete Ability Assessment was developed to assess movement abilities specific to adult athletic populations.	The authors selected movements and assessment criteria that expose deficiencies in functional movement patterns which are required to train and perform competitively in sports.
Myer et al. [102]	Tuck jump assessment	Injury risk	Self-selected	This assessment was developed to identify movement dysfunction of the lower body that may place an individual at risk of an ACL injury. Moreover, the authors proposed that its results could be used to inform resistance training programs that aim to reduce the risk of ACL injury.	The authors selected the tuck jump movement as it is an easy movement to assess in a clinical setting. The assessment criteria were ACL injury risk factors identified in previous studies of athletes.
Myer et al. [103]	Back squat assessment	Physical fitness	Self-selected	The back squat assessment was developed to be used as a screening tool to identify biomechanical deficits that may hinder optimal movement patterns in sport and physical activity.	The authors selected the back squat as it is a foundational resistance training exercise. The assessment criteria were selected by authors based on previous research about risk factors for injury in sport.
Padua et al. [106]	Landing Error Scoring System	Injury risk	Self-selected	This assessment was developed to provide a standardised tool for identifying movement patterns associated with a high risk of ACL injury during landing.	The authors self-selected the drop jump as the movement for screening and designed the assessment criteria to identify movements associated with ACL injury risk.
Padua et al. [107]	Landing Error Scoring System – Real Time	Injury risk	Re-designed	The authors wished to improve the utility of the LESS by developing a way to score it in real time.	The authors based the scoring criteria in this assessment on the biomechanical risk factors for ACL injury. How these criteria differed between the video and real time version of the LESS was not reported.
Parsonage et al. [18]	Conditioning Specific Movement Tasks	Physical fitness	Self-selected	This assessment was developed to allow the authors to assess skill across movements that are commonly executed in rugby union gym- and field-based conditioning programs.	The authors self-selected movements relevant for gym- and field-based conditioning programs in rugby.
Reid et al. [16]	Netball Movement Screening Tool	Injury risk	Self-selected	The authors wished to develop a movement screening tool that identified the movement dysfunctions that may be associated with increased injury risk in netball.	In consultation with physiotherapists and medical staff working with elite netball athletes, authors selected movements and designed criteria associated with sport performance and reduced injury risk in netball.
Rogers et al. [115]	Athlete Introductory Movement Screen	Physical fitness	Modified Delphi	The purpose of developing this screening tool was to enable a common movement assessment to be used with adolescent athletes in athlete development settings. A secondary purpose was to increase the visibility and understanding of movement competencies in entry-level adolescent athletes with aspirations to further their physical capacity via introductory strength and conditioning.	The authors selected movements to include in the screen assessment after: 1) Short-listing movements that could be used in a screening task for adolescent athletes with a low resistance training age 2) Using a systematic review [4] to guide screen development 3) Unanimous agreement of four strength and conditioning coaches of the movement selection Assessment criteria was selected from the AAA and RTSB and modified by the authors. Justifications for the modifications were reported.
Rogers et al. [116]	AAA-6	Physical fitness	Re-designed (Sensitivity)	The authors re-designed the scoring system of the AAA in an attempt to improve the sensitivity of the tool to changes in response to exercise interventions.	To increase the sensitivity of the AAA by increasing the scoring method to a Likert scale from 1 to 6.
Terry et al. [135]	Modified Musculoskeletal Readiness Tool	Injury risk	Self-selected	This tool was created as a return to duty tool for military populations (adult) that contains movement quality tests with moderate predictive validity for musculoskeletal injury with military tasks.	Six functional movements and their assessment criteria were selected by military physical therapists.
Vidal et al. [138]	Overhead Squat	Physical fitness	Self-selected	The authors wanted to assess movement dysfunction in a common movement that is in multiple screens.	The authors selected a popular functional movement used in movement quality assessments.
Woods et al. [5], Woods et al. [8]	Modified Athlete Ability Assessment	Physical fitness	Re-designed	This assessment was developed as a modification of the AAA which is reflective of the common fundamental athletic movements required to perform conditioning activities in adolescent and adult team ball sports.	The authors selected how they modified the movements from the AAA.

AAA = Athlete Ability Assessment, ACL = anterior cruciate ligament, CKCUEST = Closed Kinetic Chain Upper Extremity Stability Test, FMS = Functional Movement Screen, RTSB = Resistance Training Skills Battery, LESS = Landing Error Scoring System

#### Method of Development

In 21 studies, authors self-selected movements and assessment criteria or modified existing assessment criteria (Table 5). Four studies verified their content using expert consensus reached through a modified Delphi method. Two studies modified the scoring system of existing assessments to improve their sensitivity.

### Assessments Identified and Movements Included

Within the 113 studies reporting the *movements included* in a movement quality assessment, there were a total of 36 different assessments (Table 4). These assessments consisted of 59 movements (Table 6, Online Resource 3). Each assessment had between one and 33 *movements included*. There were 11 (31%) lower body-specific assessments and three (%) upper body-specific assessments, with all other screens assessing whole body movement quality (*N* = 22; 61%). The most commonly used assessment was the FMS (*N* = 71 studies; 63%) and a further 12 studies used modified versions of the FMS. No other assessment was used in greater than four studies. Of the 36 assessments, eight were modified variations of others. Of the *movements included*, the squat was most common (*N* = 15), followed by the lunge (*N* = 12), push up (*N* = 10), and hurdle step (*N* = 6). All other movements were used in less than five assessments (see Online Resource 3).

**Table 6:** Assessment structure of movement quality assessments including number of movements, movements included, and sets and repetitions performed during the assessment, segmental, movement and composite scoring systems and the marking approach (positive/negative)

Study	Assessment	Number of Movements	Movements	Sets and Repetitions	Segmental or Whole Body Analysis	Segmental Score	Movement Score	Composite Score	Marking Approach
Frohm et al. [54]	9 + screening battery	9	Deep squat, One-legged squat In-line lunge Active hip flexion Straight leg raises Push up Diagonal lift Seated rotation Shoulder mobility	1 × 3	Whole Body	N/A	Maximum: 3 Minimum: 1	Maximum: 27 Minimum: 9 Range: 18	Positive
Matsel et al. [95]	Arm Care Screening Tool	3	Shoulder mobility 90/90 total body rotation Lower body diagonal reach	1 × 1	Whole Body	N/A	Maximum: 1 Minimum: 0	Maximum: 3 Minimum: 0 Range: 3	Positive
Rogers et al. [115]	Athlete Introductory Movement Screen	4	Overhead squat Push up Lunge Brace with shoulder taps	2 × 4	Segmental	Maximum: 3 Minimum: 1	Maximum: 12 Minimum: 4	Maximum: 48 Minimum: 16 Range: 32	Positive
McKeown et al. [10]	Athletic Ability Assessment	9	Prone hold on hands Lateral hold on hands Overhead squat Walking lunge Single leg forward hop Lateral bound Push up Chin up	1 × 3 to maximum repetitions	Segmental	Maximum: 3 Minimum: 1	Maximum: 9 Minimum: 3	Maximum: 81 Minimum: 27 Range: 54	Positive
Myer et al. [103]	Back squat assessment	1	Back squat	1 × 10	Segmental	Maximum: 1 Minimum: 0	Maximum: 10 Minimum: 0	Maximum: 10 Minimum: 0 Range: 10	Negative
Hernandez-Garciz et al. [66]	Basic Fundamental Movement Assessment	5	Overhead squat Hurdle step Forward step-down Shoulder mobility test Active straight leg raise	1 × 2	Segmental	Maximum: 1 Minimum: 0	Maximum: 17 Minimum: 0	Maximum: 62 Minimum: 0 Range: 62	Negative
Goldbeck & Davies [60]	Closed Kinetic Chain Upper Extremity Stability Test	1	Prone hold on hands with alternate hand touch	1 x maximum repetitions	Whole Body	N/A	N/A	N/A	Positive
Parsonage et al. [18]	Conditioning Specific Movement Tasks	6	Overhead squat Romanian deadlift Single leg squat Double leg to single leg landing Sprint Countermovement jump	1 × 2	Whole Body	N/A	Maximum: 3 Minimum: 1	Maximum: 18 Minimum: 6 Range: 12	Positive
Jaffri et al. [69]	Dynamic Leap and Balance Test	1	Leap from central target to peripheral target	1 x minimum time	Whole Body	N/A	N/A	N/A	Positive
Cook et al. [11], Cook et al.[12]	Functional Movement Screen	7	Overhead squat Hurdle step In line lunge Shoulder mobility Trunk stability push up Rotary stability	1 × 3	Whole Body	N/A	Maximum: 3 Minimum: 0	Maximum: 21 Minimum: 0 Range: 21	Positive
Butler et al. [39]	Functional Movement Screen-100	7	Overhead squat Hurdle step In line lunge Shoulder mobility Trunk stability push up Rotary stability	1 × 3	Segmental	Maximum: 8 Minimum: 0	Maximum: 18 Minimum: 0	Maximum: 100 Minimum: 0 Range: 100	Positive
Hartigan et al. [65]	In Line Lunge	1	In Line Lunge	1 × 3	Whole Body	N/A	Maximum: 3 Minimum: 0	Maximum: 3 Minimum: 0 Range: 3	Positive
Padua et al. [106]	Landing Error Scoring System	1	Drop jump from a 30 cm box	1 × 3	Segmental	Maximum: 2 Minimum: 0	Maximum: 19 Minimum: 0	Maximum: 19 Minimum: 0 Range: 19	Negative
Padua et al. [107]	Landing Error Scoring System - Real Time	1	Drop jump from a 30 cm box	1 × 4	Segmental	Maximum: 2 Minimum: 0	Maximum: 15 Minimum: 0	Maximum: 15 Minimum: 0 Range: 15	Negative
Whatman et al. [141]	Lower Extremity Functional Tests	4	Bilateral small knee bend Single leg small knee bend (dominant side) Lunge (dominant side) Hop lunge (dominant side)	1 × 3	Segmental	Maximum: 3 Minimum: 0	Maximum: 21 Minimum: 0	Maximum: 84 Minimum: 0 Range: 84	Negative
Ireton et al. [6]	Modified Athletic Ability Assessment	5	Overhead squat Double lunge Single leg Romanian deadlift Push up Pull up	1 × 5 to 30	Segmental	Maximum: 3 Minimum: 1	Maximum: 9 Minimum: 3	Maximum: 45 Minimum: 15 Range: 30	Positive
Woods et al. [5, 8]	Modified Athletic Ability Assessment	4	Overhead squat Double lunge Single leg Romanian deadlift Push up	1 × 5 to 30	Segmental	Maximum: 3 Minimum: 1	Maximum: 9 Minimum: 3	Maximum: 36 Minimum: 12 Range: 24	Positive
Degot et al. [47]	Modified Closed Kinetic Chain Upper Extremity Stability Test	1	Prone hold on hands with alternate hand touch	1 x maximum repetitions	Whole Body	N/A	N/A	N/A	Positive
Terry et al. [135]	Modified Musculoskeletal Readiness Tool	7	Forward lunge Modified deep squat Closed kinetic chain upper extremity stability test	1 × 1 to maximum repetitions	Whole Body	N/A	Maximum: 2 Minimum: 0	Maximum: 17 Minimum: 0 Range: 17	Positive
Gonzalo-Skok et al. [61]	Modified Star Excursion Balance Test	5	Limb excursion in anterior, anteromedial, medial, posteomedial and posterolateral directions	1 x maximum distance	Whole Body	N/A	N/A	N/A	Positive
Kritz et al. [1]	Movement Competency Screen	6	Posture Bodyweight squat Lunge and twist Push up Bend and pull Single leg squat	1 × 1	Segmental	Maximum: 1 Minimum: 0	Maximum: 3 Minimum: 1	Maximum: 18 Minimum: 6 Range: 12	Positive
Inovero et al. [68]	Movement Competency Screen − 2	10	Squat Bilateral counter movement jump Lunge and twist Bilateral broad jump to a unilateral land Single leg squat Bilateral counter movement jump to a unilateral land Push up Explosive push up Bend and pull Bend and pull at speed	1 × 2	Segmental	Maximum: 1 Minimum: 0	Maximum: 5 Minimum: 1	Maximum: 50 Minimum: 10 Range: 40	Positive
Butowicz et al. [38]	Movement System Screening Tool	33	Overhead squat Trunk stability push up Double leg hip bridge Active hip abduction (left and right) Resisted active hip abduction (left and right) Side bridge (left and right) Side bridge hip abduction (left and right) Side bridge hip abduction resisted (left and right) Modified Thomas test (left and right) Rotary stability (left and right) Hurdle step (left and right) Active straight leg raise (left and right) Bridge leg extension (left and right) Bridge leg extension resisted (left and right) Prone hip extension (left and right) Prone hip extension arm lift (left and right) Step down (left and right) In line lunge (left and right)	1 × 3	Whole Body	N/A	Maximum: 3 Minimum: 0	Maximum: 96 Minimum: 0 Range: 96	Positive
Reid et al. [16]	Netball Movement Screening Tool	11	Bodyweight squat Lunge and twist Bend and pull Push up Single leg squat Bilateral jump and land on both legs Bilateral jump and land on single leg Broad jump Star excursion balance test in (a) anterior, (b) posterolateral and (c) posteromedial directions	1 × 6	Whole Body	As per MCS for MCS component	Maximum: 3 Minimum: 0	Maximum: 33 Minimum: 0 Range: 33	Positive
Lubans et al. [17]	Resistance Training Skills Battery	6	Squat Push up Lunge Suspended row Standing overhead press Front support with chest touches	2 × 4	Segmental	Maximum: 1 Minimum: 0	Maximum: 5 Minimum: 0	Maximum: 56 Minimum: 0 Range: 56	Positive
Glaws et al. [58]	Selective Functional Movement Assessment	10	Cervical flexion Cervical extension Cervical rotation Upper extremity pattern 1 (medial rotation, adduction, extension) Upper extremity pattern 2 (lateral rotation, abduction, flexion) Multi-segmental flexion Multi-segmental extension Multi-segmental rotation Single leg balance Overhead deep squat	1 × 1	Whole Body	N/A	Maximum: 3 Minimum: 0	Maximum: 30 Minimum: 0 Range: 30	Positive
Roush et al. [117]	Step Down Test	1	Step down off a 20 cm box	1 × 5	Segmental	Maximum: 2 Minimum: 0	Maximum: 5 Minimum: 0	Maximum: 5 Minimum: 0 Range: 5	Negative
Myer et al. [102]	Tuck Jump Assessment	1	Tuck jump	1 x maximum in 10 s	Segmental	Maximum: 1 Minimum: 0	Maximum: 10 Minimum: 0	Maximum: 10 Minimum: 0 Range: 10	Negative
Mann et al. [94]	Untitled	9	Tuck jump Overhead squat Single leg squat (left and right) Dip test (left and right) Forward lunge (left and right) Prone hold	1 × 1	Whole Body	N/A	Maximum: 3 Minimum: 1	Maximum: 27 Minimum: 9 Range: 18	Positive
Bennell et al. [35]	Weight bearing dorsiflexion	1	Ankle dorsiflexion in lunge	1 x maximum distance	Whole Body	N/A	N/A	N/A	Positive
Smith et al. [132]	Y Balance Test - Lower Quartile	3	Reach with lower leg in the anterior, posteromedial and posterolateral directions	1 × 3	Whole Body	N/A	N/A	N/A	Positive
Gorman et al. [62]	Y Balance Test - Upper Quartile	3	Reach with arm in the anterior, posteromedial and posterolateral directions	1 × 3	Whole Body	N/A	N/A	N/A	Positive

Note: MCS = Movement Competency Screen

### Scoring Systems and Criteria

The *scoring systems* of assessments followed either a Likert scale or binary outcome (Table 6, for details see Online Resource 4). Scoring was related to either certain regions of the body (*N* = 14) or to the whole body (*N* = 17). Likert scales differed between assessments and movements, with the most common being the 3-point segmental (*N* = 28; 21%) and 4-point whole body (*N* = 57; 42%) scales. The number of criteria differed between movements and assessments. The Landing Error Scoring System [106] had the greatest number of scoring criteria (*n* = 17) and the FMS-100 [39] had the greatest composite score achievable and greatest range between the lowest and highest score. Most studies scored movements across different body regions using a positive marking approach with a greater score indicating a higher quality of movement. Scoring criteria were often related to movement dysfunction, but also could be related to the number of repetitions completed, or whether pain was present during the activity. Scoring for all assessments was conducted either live or via video (or both). When scoring live, the plane of view was mostly unspecified. For video scoring, the video plane was either unspecified or in the frontal and/or sagittal plane. Overall, 13 (13%) studies specified the plane of view for assessment out of the 102 who reported assessment instructions. No standard distance from the participant was identified in the literature for live or video assessment.

### Reliability

#### Intra-rater Reliability

A total of 50 (36%) studies reported intra-rater reliability, 32 (23%) reported intra-rater reliability for composite scores, and 22 (16%) reported intra-rater reliability for each movement in their respective assessment. Results of the meta-analysis showed that intra-rater reliability for composite scores of each movement quality assessment was very high (*r* = 0.939, 95% CI 0.909–0.959). Intra-rater reliability of the assessment of a movement was moderate (K = 0.57), but varied substantially between movements (range: 0.27–0.89).

#### Inter-rater Reliability

A total of 32 (23%) studies reported inter-rater reliability, 22 (16%) reported inter-rater reliability for composite scores, and 23 (17%) reported inter-rater reliability of each movement in an assessment. Generally, inter-rater reliability was high for assessments scored using composite scores (*r* = 0.887, 95% CI 0.783–0.942). The inter-rater reliability of each movement was substantial (K = 0.63 ± 0.34), and ranged from fair agreement (Single Leg Squat: K = 0.20) to almost perfect agreement (Trunk stability push up: K = 0.89). Four studies assessed inter-rater reliability between raters of different expertise. While there was almost perfect agreement between assessments undertaken by experienced practitioners and novice practitioners, there was slight agreement between student assessors and novice practitioners. The inter-rater reliability of student assessors and expert practitioners varied between studies.

### Minimum Detectable Change

Minimum detectable change (MDC) was assessed in 13 (9%) studies, spread across eight assessments. The MDC of composite scores was low for the FMS (Live: 0.9, Video: 1.0) [26] and moderate to high for the AAA (Video: 2.9) [10], 9 + screening tool (8.3–9.5) [32] and Selective Functional Movement Assessment (3.3–9.5) [58]. The MDC for each movement in the AAA [10] and modified AAA has been reported [114]. The lowest MDC for a movement in the AAA was for the lateral hold (Left: 0.7, Right: 0.9) [10], while in the modified AAA it was for the double leg lunge (Range: 0.6–0.9) [114]. The highest MDCs were for the hop test (Left: 0.8, Right: 1.1) and Lunge test (1.0) in the AAA and the overhead squat test in the modified AAA (Range: 3.7–3.9) [10, 114].

### Technical Error

Technical error of measurement (TEM) was reported in six studies (4%). Low TEM was present in FMS assessment of adults (Maximum TEM = 0.5) [26, 105]. TEM increases when FMS movements were assessed in adolescents (Maximum TEM = 1.0) [131]. TEM has been reported for the Selective Functional Movement Assessment (1.2–2.7) [58] and Y Balance Test Lower Quarter (1.9–4.2 cm) [122] and Upper Quarter (1.8–7.6 cm) [62].

## Discussion

The aim of this study was to systematically review the (i) *developmental approach*, (ii) *movements included* and (iii) *scoring systems* used in movement quality assessments administered in athletic populations. The *reliability* of these assessments was also determined using meta-analysis and measures of error were reported. The results demonstrated that a large number of movement quality assessments have been developed for athletic populations (*n* = 36), while developmental approaches and the movements included in these assessments vary substantially. Almost all assessments used Likert scales as their scoring system, but differed in scoring criteria. The meta-analysis showed intra and inter-rater reliability of assessment composite scores were high to very high and composite scores appeared more reliable compared to movement scores. These results may guide researchers and practitioners when making informed decisions regarding the selection of a movement quality assessment.

### Developmental Approach

The results highlight that the purpose of developing movement quality assessments is related to identifying individuals at greater injury risk or identifying those who possess movement dysfunction that may hinder the development of physical fitness. These findings corroborate those of other reviews [2, 3]. Assessments have been made for adult [5, 8, 10] and adolescent [5, 8, 17, 95, 115] populations and for the sports of rugby [18], netball [16] and baseball [95]. Hence, assessment of movement quality can occur for a variety of purposes. Researchers and practitioners should consider the purpose of development of an assessment prior to its use. Best practice would be to ensure the purpose of development of an assessment aligns with the purpose of assessing movement quality in research or practice.

This review also showed that the methods of selecting movements and scoring criteria for these are prone to subjectivity. This supports the finding of a previous review which critically appraised the method of development of some movement quality assessments [2]. In that review, a limited number of studies provided rigorous justification for the composition of assessments, with only one assessment using expert consensus [2]. Coupled with the current results, this is a concern, as whilst practical measures of movement quality are convenient, they may lack the rigour required to exhibit content validity. Altogether, the varied purposes and methods used to develop movement quality assessments could explain the variation in the movements included, and the number of assessments that have been developed.

There were a limited number of assessments developed using methods with low risk of bias and expert-verification, which are tenets of content validity [147]. Examples of such assessments include the Resistance Training Skills Battery [17], Athlete Introductory Movement Screen [115], Tuck Jump Assessment [102] and the Landing Error Scoring System [106, 107]. Researchers and practitioners should attempt to use these assessments when evaluating movement quality aligned with the purposes of these assessments. Moreover, those designing novel movement quality assessments should consider using a method of development which reduces the risk of bias and improves content validity.

### Assessments Identified and Movements Included

The results demonstrated the variety of quality assessments and subsequent movements included. Nevertheless, the FMS was the most commonly reported. This popularity, in part, could be traced to its feasibility. Notably, the 4-point Likert scale to assess movement quality appears relatively easy to use, with the criteria being easily discernible. Moreover, there are clear instructions for its administration relative to other assessments [11, 12], and FMS composite scores have been associated with measures of physical fitness in youth athletes [9], and injury prognosis in athletes [148]. However, some have criticised the use of the FMS, given its low internal consistency [85] and issues with its construct validity [55, 56, 149, 150]. Based on these findings, it is recommended researchers and practitioners carefully consider the use of this assessment, appreciating both its strengths and limitations.

The results showed that whole body and lower limb movement quality can be assessed using a variety of assessments, while assessments of upper body and rotational function have received less attention. It is suggested that movements related to the lower body are favoured in movement quality assessments due to injury considerations [151]. Moreover, assessment of movement quality within the shoulder and torso regions may be difficult to quantify given the breadth of kinematic factors localised to these areas [152–155]. With that said, some assessments have been developed specific to upper body function [47, 60, 62, 95], with the most common movement being the push up. Nonetheless, the current results demonstrate the upper body is investigated at a lower rate when compared to other body regions in movement quality assessments.

### Scoring Systems and Criteria

There are distinct scoring systems and criteria for each movement quality assessment developed for athletic populations. Scoring criteria may include non-kinematic elements such as number of repetitions completed and/or the occurrence of pain during a movement. Moreover, criteria for the same movement can be different between assessments. This is likely due to the variation noted in the method of development, along with the purpose of the assessment. Indeed, while scoring criteria do not necessarily have to be the same between assessments, they should align with their purpose. A rigorous method of development could help support this process. For example, aligning scoring criteria to scientific evidence and/or expert consensus could help ensure that scoring is based on criteria that are sensitive enough to identify what they intend to.

A common criticism of current movement quality assessments is their lack of sensitivity [156], which can be a direct effect of poor scoring systems and criteria. The results of this review highlight the variation in Likert scales and scoring criteria between assessments. Moreover, the range of scores achievable are reported and assessments with low ranges may be less sensitive to changes in movement quality. A consequence of low sensitivity is that small to moderate changes in movement over time or due to intervention may be undetectable. This is a factor that may limit the utility of movement quality assessments in practice. As such, it was unsurprising to note that some assessments had been re-developed by authors to improve their sensitivity [39, 116] by increasing the range of scores achievable. To guide this re-development process, our results suggest that assessments should use Likert scales with greater than four points [39, 116], combined with movement criteria selected to identify changes in movement that can be assessed reliably.

### Reliability

While the results of this review showed movement quality composite scores were reliable, the reliability of movement scores did seem questionable. This is of concern, as while composite scores may be of use for general comparisons, the assessment of individual movements is important for a variety of reasons, such as guiding targeted exercise prescription [150]. Movements requiring the greatest amount of attention with regard to the reliability of their assessment were the lunge, single leg squat, single leg Romanian deadlift and bilateral squat. Thus, it is suggested the scoring of these movements be carefully considered moving forward to ensure greater confidence, not only in the change over time, but in the comparison between different cohorts of athletes. Results further demonstrated that rater experience implicates the reliability of movement quality assessments, reiterating the importance of using the same rater when assessing movement quality or establishing inter-rater reliability and technical error prior to interpreting the results from multiple raters.

### Limitations

While a significant effort was made to follow the PRISMA guidelines [157], reporting all results within this manuscript was challenging. Consequently, detailed result tables which report the movements included, assessment criteria and scoring systems for each assessment are included as online resources. The search terms used and inclusion and exclusion criteria were broad in an attempt to capture as much information as possible about movement quality assessment reporting in the literature. Nevertheless, 30 additional studies were identified after reference list screening which suggests that the construction of the search terms may have excluded some relevant literature from the initial search. This area of research has a number of different terms synonymous with movement quality. Whilst some of these were included in the search strategy, it is possible that more could have been included to cover this breadth of terminology.

### Future Directions

The scope of this study was intentionally broad, which resulted in an analysis of many assessments of movement quality. Therefore, this review provides an overview of the features evaluated by researchers and practitioners when selecting a movement quality assessment for their context. Based on the results of this review, the developmental approach differs between movement quality assessments and guides how an assessment is constructed. This review identified assessments developed with academic rigour that screen for movement dysfunction related to lower body injury risk (Landing Error Scoring System [106, 107], Tuck Jump Assessment [102]) and for movement patterns that may hinder participation in resistance training during adolescence (Resistance Training Skills Battery [17], Athlete Introductory Movement Screen [115]). Hence, assessors of movement quality can use these assessments with confidence as they will assess movement aligned with their purpose. Moreover, the Resistance Training Skills Battery [17] has a large range of scores achievable, assesses movements across the whole body and in different planes of motion in a segmental manner making it a comprehensive, potentially sensitive, rigourously designed assessment of movement quality designed for adolescents.

For researchers and practitioners who wish to know the most reliable assessment to use for specific populations (e.g. team sport athletes) or applications (e.g. lower body injury risk), a more critical analysis is required. The current review identifies movements that are assessed with poor reliability that may be improved by re-designing their scoring criteria. Future reviews and original research could also critically evaluate the quality of assessments developed for these purposes focusing on the discriminant ability of assessment criteria, sensitivity and interpretability of movement quality assessments. These aspects have been criticised in reviews of movement quality assessments in other domains [156] and are important considerations when selecting performance tests for practitioners [19]. The results from this line of inquiry, in combination with the results of this review, could be used to re-evaluate the assessment criteria and procedures of assessments with low content validity and reliability.

Moreover, evidence from other domains shows the associations between health outcomes and movement quality differed between assessments [13]. The relationship between movement assessments and their target application may be similarly specific in the exercise and sport sciences. Hence, the current practice of adopting assessments developed for other purposes may affect associations between movement quality and measures such as physical fitness [2, 9]. Therefore, the development of new assessments to evaluate movement quality and its association with physical fitness is warranted. When creating new assessments, developers must ensure that new assessments are designed to identify movement qualities that transfer with specificity to their desired application. Moreover, a clear rationale for movements included and their scoring criteria and expert consensus of an assessment’s structure should occur during the development process to ensure the creation of assessments that are valid and reliable.

## Conclusion

There are numerous assessments used to evaluate the movement quality of athletes with diverse developmental approaches. As a consequence, the movements included and scoring criteria of assessments vary substantially. Researchers and practitioners must carefully select the right assessment for their context. Assessments which exhibit content validity can be selected with confidence that their contents align with their purpose. The reliability of composite scores is very high and guidelines for reporting reliability in movement quality literature are proposed. Altogether, these results could be used to guide the choice of assessment or inform the design of new assessments that consider the developmental approach, movements included, scoring criteria and their subsequent reliability in athletic populations.

## Electronic Supplementary Material

Below is the link to the electronic supplementary material.

Supplementary Material 1

Supplementary Material 2

Supplementary Material 3

Supplementary Material 4

## Data Availability

All data supporting the findings of this study are reported within this manuscript or its online resources.

## Abbreviations

AAA: Athletic Ability Assessment
FMS: Functional Movement Screen
ICC: intraclass correlation coefficients
MDC: minimum detectable change
TEM: technical error of measurement
