# Supplementary material for: The Development and Content of Movement Quality Assessments in Athletic Populations: A Systematic Review and Multilevel Meta-Analysis
Source: Sports Med Open. 2025 Jan 23;11:7. doi: 10.1186/s40798-025-00813-0 (PMC11757847; doi:10.1186/s40798-025-00813-0)
Supplement: Supplementary file 2 — Supplementary Material 2 [file 40798_2025_813_MOESM2_ESM.pdf]

Online Resource 2 The study quality of each included study in the manuscript "*The development and content of movement quality assessments in athletic populations: a systematic review and multilevel meta-analysis*".

Study	Hypothesis described	Outcomes described	Participant characteristics described	Main findings described	Random variability (error) described	Probability of main outcomes (p-value)	Participants appropriate for question	Blinding of outcomes for participants	Blinding of outcomes for assessors	P-hacking evidence?	Statistical tests appropriate for aims	Outcome measures used accurate	Adjustment for covariates	Power calculation/adequacy	Study Quality (/14)	Study Quality Descriptor
Alkhathami 2021	Yes	Yes	Yes	Yes	Yes	Yes	Yes	No	No	No	Yes	Yes	No	Yes	11	Good
Armstrong 2018	Yes	Yes	Yes	Yes	Yes	Yes	Yes	No	No	No	Yes	Yes	No	Yes	11	Good
Armstrong 2018	Yes	Yes	Yes	Yes	Yes	Yes	Yes	No	No	No	Yes	Yes	No	Yes	11	Good
Armstrong 2020	Yes	Yes	Yes	Yes	Yes	Yes	Yes	No	No	No	Yes	Yes	No	Yes	11	Good
Atalay 2018	Yes	Yes	Yes	Yes	No	Yes	Yes	No	No	No	Yes	Yes	No	Yes	10	Good
Okada 2011	Yes	Yes	Yes	Yes	No	Yes	Yes	No	No	No	Yes	Yes	No	Yes	10	Good
Bakalar 2020	Yes	Yes	Yes	Yes	No	Yes	Yes	No	No	No	Yes	Yes	No	No	9	Fair
Bakken 2017	Yes	Yes	Yes	Yes	Yes	Yes	Yes	No	No	No	Yes	Yes	Yes	Yes	12	Good
Barnett 2015	Yes	Yes	Yes	Yes	Yes	Yes	Yes	No	Yes	No	Yes	Yes	Yes	Yes	13	Good
Bennett 2022	Yes	Yes	Yes	Yes	Yes	No	Yes	No	No	No	Yes	Yes	No	Yes	10	Good
Borms 2018	Yes	Yes	Yes	Yes	Yes	No	Yes	No	No	No	Yes	Yes	Yes	Yes	11	Good
Bullock 2017	Yes	Yes	No	Yes	Yes	Yes	Yes	No	No	No	Yes	Yes	Yes	Yes	11	Good

Butler 2012	Yes	Yes	Yes	Yes	No	No	Yes	Yes	No	No	Yes	Yes	No	Yes	10	Good
Butowicz 2020	Yes	Yes	Yes	Yes	Yes	Yes	Yes	No	No	No	Yes	Yes	Yes	Yes	12	Good
Campa 2019	Yes	Yes	Yes	Yes	No	Yes	Yes	No	No	No	Yes	Yes	No	Yes	10	Good
Chang 2020	Yes	Yes	Yes	Yes	No	Yes	Yes	No	No	No	Yes	No	No	Yes	9	Fair
Chapman 2014	Yes	Yes	Yes	Yes	No	Yes	Yes	No	No	No	Yes	Yes	No	Yes	10	Good
Chimera 2017	Yes	Yes	Yes	Yes	Yes	Yes	Yes	No	No	No	Yes	Yes	No	Yes	11	Good
Clifton 2015	Yes	Yes	Yes	Yes	Yes	Yes	Yes	No	No	No	Yes	Yes	No	Yes	11	Good
Conkin 2020	Yes	Yes	Yes	Yes	No	No	Yes	No	No	No	Yes	Yes	No	Yes	9	Fair
Cook 2014	Yes	Yes	No	Yes	No	No	No	No	No	No	No	Yes	No	No	5	Poor
Cook 2014	Yes	Yes	No	Yes	No	No	No	No	No	No	No	Yes	No	No	5	Poor
Davis 2020	Yes	Yes	No	Yes	No	Yes	Yes	No	No	No	No	Yes	No	Yes	8	Fair
deOliveira 2017	Yes	Yes	Yes	Yes	Yes	Yes	Yes	No	No	No	Yes	Yes	No	No	10	Good
Dobbs 2021	Yes	Yes	Yes	Yes	Yes	Yes	Yes	No	No	No	Yes	Yes	No	Yes	11	Good
Domaradzki 2023	Yes	Yes	Yes	Yes	Yes	Yes	Yes	No	No	No	Yes	Yes	No	Yes	11	Good
Edis 2021	Yes	Yes	Yes	Yes	No	No	Yes	No	No	No	Yes	Yes	No	Yes	9	Fair
Ferreira 2021	Yes	Yes	Yes	Yes	Yes	Yes	Yes	No	No	No	Yes	Yes	No	Yes	11	Good
Fox 2014	Yes	Yes	Yes	Yes	Yes	Yes	Yes	No	No	No	Yes	Yes	Yes	Yes	12	Good
Frohm	Yes	Yes	Yes	Yes	No	No	Yes	No	No	No	Yes	Yes	No	Yes	9	Fair

2011																
Frost 2012	Yes	Yes	Yes	Yes	Yes	Yes	Yes	Yes	No	No	Yes	Yes	Yes	Yes	13	Good
Frost 2015	Yes	Yes	Yes	Yes	Yes	Yes	Yes	Yes	No	No	Yes	Yes	Yes	Yes	13	Good
Garrett 2018	Yes	Yes	Yes	Yes	Yes	No	Yes	Yes	No	No	Yes	Yes	Yes	Yes	12	Good
Gavigan 2022	Yes	Yes	Yes	Yes	No	No	Yes	No	No	No	Yes	Yes	No	Yes	9	Fair
Gnacinski 2016	Yes	Yes	Yes	Yes	Yes	No	Yes	No	No	No	Yes	Yes	Yes	Yes	11	Good
Glass 2017	Yes	Yes	Yes	Yes	Yes	No	Yes	No	No	No	Yes	Yes	Yes	Yes	11	Good
Glaws 2014	Yes	Yes	Yes	Yes	No	No	Yes	Yes	Yes	No	Yes	Yes	No	Yes	11	Good
Goldbeck 2000	Yes	Yes	Yes	Yes	Yes	No	Yes	No	No	No	Yes	Yes	Yes	No	10	Good
Gonzalo-Skok 2015	Yes	Yes	Yes	Yes	No	Yes	Yes	No	No	No	Yes	Yes	Yes	Yes	11	Good
Gorman 2012	Yes	Yes	Yes	Yes	No	No	Yes	No	No	No	Yes	Yes	No	No	8	Fair
Gribble 2013	Yes	Yes	Yes	Yes	Yes	Yes	Yes	No	Yes	No	Yes	Yes	Yes	Yes	13	Good
Gulgin 2012	Yes	Yes	Yes	Yes	Yes	No	Yes	No	No	No	Yes	Yes	No	No	9	Fair
Harshbarger 2018	Yes	Yes	Yes	Yes	No	No	Yes	No	Yes	No	Yes	Yes	No	Yes	10	Good
Hartigan 2014	Yes	Yes	Yes	Yes	No	Yes	Yes	No	No	No	Yes	Yes	No	No	9	Fair
Hernandez-Garcia 2020	Yes	Yes	Yes	Yes	No	No	Yes	No	No	No	No	Yes	No	No	7	Poor

Hollstadt 2020	Yes	Yes	Yes	Yes	No	No	Yes	No	No	No	No	Yes	Yes	No	8	Fair
Inovero 2016	Yes	Yes	Yes	Yes	No	No	Yes	No	No	No	Yes	Yes	No	No	8	Fair
Ireton 2019	Yes	Yes	Yes	Yes	Yes	Yes	Yes	No	No	No	Yes	Yes	Yes	Yes	12	Good
Jaffri 2017	Yes	Yes	Yes	Yes	Yes	Yes	Yes	No	No	No	Yes	Yes	No	Yes	11	Good
Kara 2020	Yes	Yes	Yes	Yes	No	No	Yes	No	No	No	Yes	Yes	No	Yes	9	Fair
Kara 2021	Yes	Yes	Yes	Yes	No	No	Yes	No	No	No	Yes	No	No	Yes	8	Fair
Kazman 2014	Yes	Yes	Yes	Yes	Yes	No	Yes	Yes	Yes	No	Yes	Yes	Yes	Yes	13	Excellent
Kelleher 2018	Yes	Yes	Yes	Yes	Yes	Yes	Yes	Yes	Yes	No	Yes	Yes	Yes	Yes	14	Excellent
Kuzuhara 2018	Yes	Yes	Yes	Yes	No	No	Yes	No	No	No	Yes	Yes	No	Yes	9	Fair
Koehle 2016	Yes	Yes	Yes	Yes	Yes	Yes	Yes	Yes	Yes	No	Yes	Yes	Yes	Yes	14	Excellent
Kokstejn 2019	Yes	Yes	Yes	Yes	No	Yes	Yes	No	No	No	No	Yes	No	Yes	9	Fair
Kozlenia 2020	Yes	Yes	Yes	Yes	No	No	Yes	No	No	No	Yes	Yes	Yes	Yes	10	Good
Kozlenia 2021	Yes	Yes	No	Yes	Yes	Yes	Yes	No	No	No	Yes	Yes	Yes	Yes	11	Good
Kramer 2019	Yes	Yes	Yes	Yes	No	No	Yes	No	No	No	Yes	Yes	Yes	Yes	10	Good
Kraus 2019	Yes	Yes	No	Yes	No	No	Yes	No	No	No	Yes	Yes	No	Yes	8	Fair
Krysak 2019	Yes	Yes	Yes	Yes	No	Yes	Yes	No	No	No	Yes	Yes	Yes	Yes	11	Good
Lee 2015	Yes	Yes	Yes	Yes	Yes	No	Yes	No	No	No	Yes	Yes	No	No	9	Fair

Lee 2018	Yes	Yes	Yes	Yes	Yes	Yes	Yes	No	No	No	Yes	Yes	Yes	Yes	12	Good
Lee 2019	Yes	Yes	Yes	Yes	Yes	Yes	Yes	No	No	No	Yes	Yes	Yes	Yes	12	Good
Leeder 2016	Yes	Yes	Yes	Yes	No	No	Yes	No	No	No	No	Yes	No	No	7	Poor
Li 2015	Yes	Yes	No	Yes	Yes	Yes	Yes	Yes	No	No	Yes	Yes	Yes	Yes	12	Good
Liang 2019	Yes	Yes	Yes	Yes	Yes	Yes	Yes	No	No	No	No	Yes	No	Yes	10	Good
Lisman 2013	Yes	Yes	Yes	Yes	Yes	Yes	Yes	No	No	No	No	Yes	No	Yes	10	Good
Lloyd 2015	Yes	Yes	Yes	Yes	Yes	Yes	Yes	No	No	No	Yes	Yes	Yes	Yes	12	Good
Lockie 2015c	Yes	Yes	Yes	Yes	No	No	Yes	No	No	No	Yes	Yes	No	Yes	9	Fair
Lockie 2015b	Yes	Yes	Yes	Yes	Yes	No	Yes	No	No	No	Yes	Yes	Yes	No	10	Good
Lockie 2015a	Yes	Yes	Yes	Yes	Yes	No	Yes	No	No	No	Yes	Yes	No	Yes	10	Good
Loudon 2014	Yes	Yes	Yes	Yes	Yes	Yes	Yes	No	No	No	Yes	Yes	Yes	Yes	12	Good
Lubans 2014	Yes	Yes	Yes	Yes	Yes	Yes	Yes	No	No	No	Yes	Yes	Yes	Yes	12	Good
Magyari 2017	Yes	Yes	Yes	Yes	No	No	Yes	No	No	No	Yes	Yes	Yes	No	9	Fair
Mann 2022	Yes	Yes	Yes	Yes	No	No	Yes	Yes	No	No	Yes	Yes	Yes	Yes	11	Good
Matsel 2021	Yes	Yes	Yes	Yes	Yes	No	Yes	No	Yes	No	Yes	Yes	Yes	Yes	12	Good
McCann 2017	Yes	Yes	Yes	Yes	Yes	Yes	Yes	No	No	No	Yes	Yes	No	Yes	11	Good

McKeown 2014	Yes	Yes	No	Yes	Yes	Yes	Yes	No	No	No	Yes	Yes	Yes	No	10	Fair
Milbank 2016	Yes	Yes	No	Yes	No	Yes	Yes	No	No	No	Yes	Yes	Yes	Yes	10	Fair
Miller 2019	Yes	Yes	Yes	Yes	No	No	Yes	No	No	No	Yes	Yes	No	Yes	9	Fair
Minick 2010	Yes	Yes	No	Yes	No	No	Yes	No	No	No	Yes	Yes	No	Yes	8	Fair
Misegades 2020	Yes	Yes	Yes	Yes	No	Yes	Yes	No	No	No	Yes	Yes	No	No	9	Fair
Mu 2022	Yes	Yes	Yes	Yes	No	No	Yes	No	No	No	Yes	Yes	No	Yes	9	Fair
Myer 2008	Yes	Yes	No	Yes	No	No	Yes	No	No	No	Yes	Yes	No	No	7	Poor
Myer 2014	Yes	Yes	No	Yes	No	No	Yes	No	No	No	Yes	Yes	No	No	7	Poor
Okely 2001	Yes	Yes	Yes	Yes	No	Yes	Yes	No	No	No	Yes	Yes	Yes	Yes	11	Good
Onate 2012	Yes	Yes	Yes	Yes	Yes	No	Yes	No	No	No	Yes	Yes	No	Yes	10	Good
Parchmann 2011	Yes	Yes	Yes	Yes	No	No	Yes	No	No	No	Yes	Yes	No	Yes	9	Fair
Padua 2009	Yes	Yes	Yes	Yes	Yes	Yes	Yes	No	No	No	Yes	Yes	Yes	Yes	12	Good
Padua 2011	Yes	Yes	Yes	Yes	Yes	No	Yes	No	No	No	Yes	Yes	No	Yes	10	Good

Parenteau 2014	Yes	Yes	Yes	Yes	Yes	No	Yes	No	Yes	No	Yes	Yes	No	Yes	11	Good
Parsonage 2014	Yes	Yes	Yes	Yes	Yes	Yes	Yes	No	No	No	Yes	Yes	Yes	No	11	Good
Pichardo 2019	Yes	Yes	Yes	Yes	No	No	Yes	No	No	No	Yes	Yes	No	No	8	Fair
Popchak 2021	Yes	Yes	Yes	Yes	Yes	No	Yes	No	No	No	Yes	Yes	No	Yes	10	Good
Pullen 2022	Yes	Yes	Yes	Yes	Yes	No	Yes	No	No	No	Yes	Yes	Yes	Yes	11	Good
Rafnsson 2019	Yes	Yes	No	No	Yes	No	Yes	No	No	No	Yes	Yes	No	Yes	8	Fair
Reid 2015	Yes	Yes	Yes	Yes	Yes	Yes	Yes	No	No	No	Yes	Yes	No	No	10	Fair
Roger 2019	Yes	Yes	Yes	Yes	Yes	Yes	Yes	No	No	No	Yes	Yes	Yes	No	11	Good
Rogers 2019	Yes	Yes	Yes	Yes	Yes	No	Yes	No	No	No	Yes	Yes	No	No	9	Fair
Rogers 2021	Yes	Yes	Yes	Yes	Yes	Yes	Yes	No	No	No	Yes	Yes	Yes	Yes	12	Good
Roush 2010	Yes	Yes	Yes	Yes	No	Yes	Yes	No	No	No	Yes	Yes	No	Yes	10	Good
Rowell 2021	Yes	Yes	Yes	Yes	No	Yes	Yes	No	No	No	Yes	Yes	Yes	Yes	11	Good
Rowan 2015	Yes	Yes	Yes	Yes	No	No	Yes	No	No	No	Yes	Yes	No	No	8	Fair
Schneiders 2011	Yes	Yes	Yes	Yes	No	No	Yes	No	No	No	Yes	Yes	No	Yes	9	Fair
Schwiertz 2019	Yes	Yes	Yes	Yes	Yes	No	Yes	No	No	No	Yes	Yes	Yes	No	10	Good
Shaffer 2013	Yes	Yes	Yes	Yes	Yes	No	Yes	No	No	No	Yes	Yes	No	No	9	Fair
Shojaedin 2014	Yes	Yes	Yes	Yes	No	No	Yes	No	No	No	Yes	Yes	No	Yes	9	Fair

Shultz 2013	Yes	Yes	Yes	Yes	Yes	No	Yes	No	No	No	Yes	Yes	No	Yes	10	Good
Sikora 2022	Yes	Yes	Yes	Yes	No	Yes	Yes	No	No	No	Yes	Yes	Yes	Yes	11	Good
Silva 2017	Yes	Yes	Yes	Yes	No	No	Yes	No	No	No	Yes	Yes	No	Yes	9	Fair
Silva 2018	Yes	Yes	Yes	Yes	No	No	Yes	No	No	No	Yes	Yes	No	Yes	9	Fair
Silva 2019a	Yes	Yes	Yes	Yes	No	No	Yes	No	No	No	Yes	Yes	Yes	Yes	10	Good
Silva 2019b	Yes	Yes	Yes	Yes	Yes	No	Yes	No	No	No	Yes	Yes	No	No	9	Fair
Smith 2013	Yes	Yes	Yes	Yes	Yes	No	Yes	No	No	No	Yes	Yes	No	No	9	Fair
Smith 2017	Yes	Yes	Yes	Yes	No	No	Yes	No	No	No	Yes	Yes	No	Yes	9	Fair
Smith 2018	Yes	Yes	Yes	Yes	Yes	No	Yes	No	No	No	Yes	Yes	No	Yes	10	Good
Sommerfield 2022	Yes	Yes	Yes	Yes	Yes	No	Yes	No	No	No	Yes	Yes	No	Yes	10	Good
Stepinski 2020	Yes	Yes	Yes	Yes	No	No	Yes	No	No	No	Yes	Yes	No	No	8	Fair
Terry 2018	Yes	Yes	Yes	Yes	No	No	Yes	No	No	No	Yes	Yes	No	Yes	9	Fair
Teyhen 2014	Yes	Yes	Yes	Yes	No	Yes	Yes	No	No	No	Yes	Yes	Yes	Yes	11	Good
Venter 2017	Yes	Yes	Yes	Yes	No	Yes	Yes	No	No	No	Yes	Yes	No	Yes	10	Good
Vidal 2018	Yes	Yes	Yes	Yes	No	Yes	Yes	No	No	No	Yes	Yes	No	Yes	10	Good
Waldron 2016	Yes	Yes	Yes	Yes	No	No	Yes	No	No	No	Yes	Yes	No	No	8	Fair

Warshaw 2018	Yes	Yes	No	Yes	No	Yes	Yes	No	No	No	Yes	Yes	No	Yes	9	Fair
Whatman 2012	Yes	Yes	Yes	Yes	Yes	No	Yes	Yes	No	No	No	Yes	Yes	Yes	11	Good
Whiteside 2016	Yes	Yes	Yes	Yes	No	No	Yes	No	No	No	Yes	Yes	No	No	8	Fair
Willigenburg 2017	Yes	Yes	Yes	Yes	No	Yes	Yes	Yes	No	No	Yes	Yes	Yes	Yes	12	Good
Woods 2016	Yes	Yes	Yes	Yes	No	No	Yes	Yes	No	No	Yes	Yes	Yes	Yes	11	Good
Woods 2018	Yes	Yes	Yes	Yes	No	Yes	Yes	Yes	No	No	Yes	Yes	Yes	Yes	12	Good
Zalai 2015	Yes	Yes	Yes	Yes	No	Yes	Yes	No	No	No	Yes	Yes	No	No	9	Fair
Zhang 2022	Yes	Yes	Yes	Yes	No	No	Yes	No	No	No	Yes	Yes	No	Yes	9	Fair
Zou 2016	Yes	Yes	Yes	Yes	No	No	Yes	No	No	No	Yes	Yes	No	No	8	Fair
