# Supplementary material for: The Development and Content of Movement Quality Assessments in Athletic Populations: A Systematic Review and Multilevel Meta-Analysis
Source: Sports Med Open. 2025 Jan 23;11:7. doi: 10.1186/s40798-025-00813-0 (PMC11757847; doi:10.1186/s40798-025-00813-0)
Supplement: Supplementary file 3 — Supplementary Material 3 [file 40798_2025_813_MOESM3_ESM.pdf]

Online Resource 3 The movements and associated assessments within the manuscript "*The development and content of movement quality assessments in athletic populations: a systematic review and multilevel meta-analysis*".

Movement	Assessment
90/90 Total Body Rotation	Arm Care Screen
Active Cervical Extension	Selective Functional Movement Assessment
Active Cervical Flexion	Selective Functional Movement Assessment
Active Hip Flexion	9+ Screening Battery
Active straight leg raise	Basic Fundamental Movement Assessment, FMS, FMS Lower Extremity, Modified FMS, Movement System Screening Tool, NMST
Bend-and-pull	MCS, NMST
Broad Jump	NMST
Cervical Rotation Side-Bend	Selective Functional Movement Assessment
Chin Up	AAA-6, AAA, Modified AAA
CKCUEST	CKCUEST, Mod-CKCUEST, Musculoskeletal Readiness Screening Tool, Movement System Screening Tool
Countermovement Jump	CSMT, NMST
Clinical Core Control tests (3 Cs)	Movement System Screening Tool
Diagonal Lift (Bird-Dog)	9+ Screening Battery
Double leg lowering test	Movement System Screening Tool
Double Leg Jump, Single Leg Land	CSMT, NMST
Drop Jump	Landing Error Scoring System
Dynamic leap and balance	Dynamic leap and balance test
Feagin Hop (SL Vertical Hop)	Musculoskeletal Readiness Screening Tool
Forward step-down test	Musculoskeletal Readiness Screening Tool, Movement System Screening Tool, Step Down Test
Glenohumeral internal rotation deficit	Movement System Screening Tool
Hop Lunge	Lower Extremity Functional Test
Hurdle step	FMS, FMS-LE, Modified FMS, Modified FMS, Movement System Screening Tool
Lateral Bound	AAA
Lateral hold on hands	AAA, Movement System Screening Tool
Lateral step down test	Lateral step down test
Lower Body Diagonal Reach (Posteromedial Reach)	Arm Care Screen
Lunge	9+ Screening Battery, AAA-6, AIMS, AAA, FMS, FMS-LE, Lower Extremity Functional Tool, Modified AAA, Modified FMS, Modified FMS, MCS, RTSB, NMST
Medial Rotation Extension	Selective Functional Movement Assessment
Multi-segmental Extension	Selective Functional Movement Assessment

Multi-segmental Flexion	Selective Functional Movement Assessment
Multi-segmental Rotation	Selective Functional Movement Assessment
One-Legged Squat Test	9+ Screening Battery, AAA, MCS, CSMT, NMST
Prone hold	AAA
Front support brace with shoulder touches	AIMS, Resistance Training Skills Battery
Push up	9+ Screening Battery, AAA-6, AAA, AIMS, Modified AAA, MCS, RTSB, NMST
Rotary Stability	FMS, Modified FMS, Movement System Screening Tool
Romanian Deadlift	CSMT
Scapular dyskinesis	Movement System Screening Tool
Seated Rotation	9+ Screening Battery
Shoulder Clearing Test	FMS
Shoulder Mobility, Reciprocal Shoulder Mobility	9+ Screening Battery, Arm Care Screen, FMS, Movement System Screening Tool
Single Leg Forward Hop	AAA
Single Leg Romanian Deadlift	AAA-6, Modified AAA
Single Leg Stance (eyes open, then closed)	Selective Functional Movement Assessment
Small knee bend, Single leg knee bend	Lower Extremity Functional Test
Spinal Extension Clearance Exam	FMS, Movement System Screening Tool
Spinal flexion clearance exam	FMS, Movement System Screening Tool
Sprint Run	CSMT
Squat	9+ Screening Battery, AAA-6, AAA, AIMS, BSA, FMS, FMS-LE, Modified AAA, Modified FMS, Modified FMS, Musculoskeletal Readiness Screening Tool, MCS, RTSB, SFMS, CSMT, NMST
Standing Overhead Press	Resistance Skills Training Battery
Standing Posture Assessment	Movement Competency Screen
Straight leg raises	9+ Screening Battery,
Suspended Row	Resistance Skills Training Battery
Trunk stability push up	FMS, Modified FMS, Modified FMS
Tuck Jump Assessment	Tuck Jump Assessment, Musculoskeletal Readiness Screening Tool
Unilateral hip bridge endurance	Movement System Screening Tool
Unilateral Wall Sit Hold	Musculoskeletal Readiness Screening Tool
Upper Extremity Pattern 1	Selective Functional Movement Assessment
Upper Extremity Pattern 2: Lateral Rotation Extension	Selective Functional Movement Assessment

AAA = Athlete Ability Assessment, AIMS = Athlete Introductory Movement Screen, CKCUEST = Closed Kinetic Chain Upper Extremity Stability Test, CSMT = Conditioning Specific Movement Test, FMS = Functional Movement Screen, LESS = Landing Error Scoring System, LQ = Lower Quarter, MCS=Movement Competency Screen, N/A = not applicable, NMST = Netball Movement Screening Tool, UQ = Upper Quarter, YBT = Y Balance Test.
