# Supplementary material for: The Development and Content of Movement Quality Assessments in Athletic Populations: A Systematic Review and Multilevel Meta-Analysis
Source: Sports Med Open. 2025 Jan 23;11:7. doi: 10.1186/s40798-025-00813-0 (PMC11757847; doi:10.1186/s40798-025-00813-0)
Supplement: Supplementary file 4 — Supplementary Material 4 [file 40798_2025_813_MOESM4_ESM.pdf]

Online Resource 4 Movement, screen, instructions for movement and scoring system and criteria within the manuscript "*The development and content of movement quality assessments in athletic populations: a systematic review and multilevel meta-analysis*".

Movement	Screen	Instructions for Movement	Criteria	Scoring System
Squat	9+ screening battery	Overhead with PVC pipe<br>A 0.02 m board is placed under the heels. Parallel feet shoulder width apart with 90° of the elbows as pre-starting position. Straighten the arms over the head with elbows extended. Keep straight arms over head, upper body should be kept in a vertical position while doing a slow squat down to a position where femur is below horizontal	3 points: All criteria have to be fulfilled (0.02 m board)<br>Straight line hip, knee and foot<br>Parallel feet and heels kept on the board throughout the motion<br>Femur below horizontal<br>Arms parallel to ears<br>The pole is behind the toes<br><br>2 points: This criterion has to be fulfilled (0.04 m board) Same criteria as for 3 points except the use of a 0.04 m board<br><br>1 point: One or more of the following criteria have to be fulfilled (0.04 m) No straight line hip, knee and foot The feet are not parallel through the motion Femur is not below horizontal Arms are not parallel with the ears The pole is not behind the toes	Likert 1 to 3
	AAA-6	Overhead with wooden dowel<br>To standardize grip width on the wooden dowel, participants are instructed to rest the dowel on their head and attain a grip width, which results in a right angle at the elbow (as per the Functional Movement Screen). Participants are then instructed to press the dowel overhead until elbows are straight.<br><br>1. Squat to the lowest position possible.<br>2. Keep the dowel in line with the back of your head.<br>3. Keep your arms straight throughout the squat.<br>4. Perform 5 repetitions.	Deviation from the following criteria were assessed using a 5-point likert scale. The scale was anchored at the following points:<br>1 = very large<br>2 = large<br>3 = moderate<br>4 = small<br>5 = very small<br>6 = criteria fulfilled<br><br>CRITERIA:<br>Upper Quadrant – stick above head and feet<br>Triple Flexion – Femur parallel<br>Trunk control – Neutral trunk	Likert 1 to 6
	Athlete Ability Assessment	Overhead with 10kg barbell x 5 repetitions<br>Athletes were given specific instructions and demonstration on how to correctly perform each movement, including a verbal description of the scoring criteria verbatim from the scoring criteria.	CRITERIA:<br>Hands/Bar overhead<br>3 – Maintains bar overhead with appropriate shoulder/thoracic extension & trunk angle with no rotation<br>2 – Bar over mid-foot but incorrect movement patterning<br>1 – Excessive or inappropriate trunk inclination<br><br>Hip/Knee/Ankle Alignment<br>3 – Perfect alignment and control of hip/knee/ankle throughout every rep<br>2 – Inconsistent form with some perfect reps OR minor misalignment on all repetitions<br>1 – Unable to attain correct position<br><br>Depth	Likert 1 to 3

			3 – Hip below knee (below parallel) while maintaining neutral spine for all repetitions<br>2 – Depth below parallel for some but not all reps<br>1 – Not able to achieve required depth for any reps	
	Athlete Introductory Movement Screen	To standardise delivery and technical instruction across testing sessions, athletes viewed pre-recorded video demonstrations in which the movements were presented and desired movement patterns described using the key points from the scoring criteria. Following the first viewing, athletes were permitted to practise four to five repetitions of the movements and then re-watch the demonstration video.	2 x 4 repetitions<br>CRITERIA:<br>Heels<br>3 – Heels remains on the ground throughout 4 consecutive repetitions<br>2 – 3 appropriate repetitions<br>1 – 2 or less appropriate repetitions<br><br>Depth<br>3 – Thighs are at least parallel to the floor at the bottom of the movement throughout 4 consecutive repetitions<br>2 – 3 appropriate repetitions; OR near parallel for all repetitions<br>1 – 2 or less appropriate repetitions<br><br>Bar (dowel) and trunk position<br>3 – Maintains bar overhead with appropriate shoulder/thoracic extension & trunk angle without rotation, throughout 4 consecutive repetitions<br>2 – 3 appropriate repetitions; OR bar positions appropriate but minor deviation from appropriate thoracic extension & trunk angle on all repetitions; OR poor bar position but with appropriate thoracic extension and trunk angle on all repetitions<br><br>Frontal plane alignment<br>3 – Appropriate alignment, symmetry and control of hip/knee/ankle, including thighs, move symmetrically throughout 4 consecutive repetitions.<br>2 – 3 appropriate repetitions; OR minor misalignment and/or asymmetry on all repetitions<br>1 – 2 or less appropriate repetitions	Likert 1 to 3
	Back Squat Assessment	A dowel is held in back squat position with forearms parallel to torso and upper back musculature contracted. Feet are placed with heels shoulder width apart and toes forward or slightly outward. Please stand upright with feet shoulder width apart. Squat down until the top of your thighs are at least parallel to the ground, and then return to the initial starting position. Perform 10 continuous repetitions at a consistent, moderate pace or until you are instructed to stop.	1. Head Position<br>- chin in neutral position<br>- point of focus, gaze straight ahead or upwards<br>2. Thoracic Position<br>- slightly extended and held rigid. Scapulae retracted and depressed, shoulders retracted.<br>3. Trunk Position<br>- neutral alignment<br>- stable without wavering<br>- slight lordotic curve<br>4. Hip Position<br>- square, stable hips<br>- symmetrical position of the femur<br>- squat depth determined on the position of the hips<br>5. Frontal Plane Knee Alignment	Deficit marked if individual fails to demonstrate desired technique in 2 repetitions

			- Knees track over toes<br>- absence of knee displacement laterally and medially<br>- medial aspect of knee not cross lateral malleolus<br>- lateral aspect of knee not cross medial malleolus<br>6. Tibial Translational Angle<br>- positive shin angle<br>- tibial angle in parallel with upright trunk<br>7. Foot Position<br>- Feet are stable and planted firmly in the ground<br>- Weight through heel and lateral foot<br>8. Descent Position<br>- breaking at the hips<br>- maintain an upright torso throughout descent with vertical distance through the shoulder/hips constant<br>9. Depth<br>- tops of thighs parallel to the ground without disjointed deviations noted at the knee, ankle or hips<br>- feet entirely on the ground<br>10. Ascent<br>- torso upright during the ascent phase<br>- shoulders and hips rise at the same pace and difference in vertical height of the shoulders and hips remain constant	
	Basic Functional Assessment Protocol	Overhead<br>Separate the feet (barefoot) to be shoulder-width apart<br>• Place the second toe facing forward in line with your knee<br>• Lift arms up, as if you would like to touch the ceiling (or the sky)<br>• When I say "ready, go", crouch your bottom down as much as you can to the ground<br>• You will perform two repetitions for each side<br>If the subject asks "is it squat?" you have to answer: "you must lower your bottom to the ground as much as possible."	VIEW: FRONT POSITION<br>External Rotation Foot<br>- Turning the foot on the longitudinal axis until the phalanges of the second right/left toe are oriented in a lateral direction<br>Internal Rotation Foot<br>- Turning the foot on the longitudinal axis until the phalanges of the second right/left toe are oriented in the medial direction<br>Knee valgus<br>- Displacement of the right/left knee in the front plane during movement, so that the distance between the two knees is reduced by staying closer to the midline of the body at the end of the movement<br>Knee varus<br>- Displacement of the right/left knee in the front plane during movement, so that the distance between the two knees is increased to the midline of the body at the end of the movement<br>Thorax Rotation<br>- Rotation of the trunk to the left/right of the observer<br><br>VIEW: BACK POSITION<br>Foot pronation<br>- A fall of the plantar arch is observed in the right/left midfoot area<br>Foot supination<br>- Overly pronounced right/left midfoot plant arch	Pass/Fail – 1 point for a "Yes" answer to the descriptors in the criteria

			Asymmetrical distribution of the hip<br>- Displacement of the pelvis in the frontal plane towards the right/left leg<br><br>VIEW: SAGITTAL POSITION<br>Heels lift<br>- The right/left heel loses contact with the support surface<br><br>Lumbo-pelvis dissociation loss<br>- Lumbar neutral curvature disappears<br><br>Excess lumbar lordosis<br>- The concavity of the lumbar curve increases too much during movement<br><br>Excess thoracic<br>- The concavity of the dorsal curve increases too much during movement<br><br>Arms fall to the front<br>- The right/left hand fingers pass the vertical line drawn from the toes, so that the right/left arm is not aligned with the trunk<br><br>Cervical extension<br>- The concavity of the cervical curve increases too much during movement<br><br>Cervical Flexion<br>- The concavity, of the cervical curve is lost too much during movement, the face is oriented in a flow direction	
	Functional Movement Screen<br><br>Modified Functional Movement Screen (1)<br><br>Modified Functional Movement Screen (2)	The individual assumes the starting position by placing his/her feet approximately shoulder width apart and the feet aligned in the sagittal plane. The individual then adjusts their hands on the dowel to assume a 90-degree angle of the elbows with the dowel overhead. Next, the dowel is pressed overhead with the shoulders flexed and abducted, and the elbows extended, so that the dowel is directly overhead. The individual is then instructed to descend as far as they can into a squat position while maintaining an upright torso, keeping the heels and the dowel in position. Hold the descended position for a count of one, and then return to the starting position. As many as three repetitions may be performed. If the criteria for a score of "3" is not achieved, the athlete is then asked to perform the test with a 2x6 block under the heels.	0 – movement causes pain<br>1 – unable to complete the movement or assume the position required to perform the movement<br>2 – able to complete the movement but must compensate (e.g. squat with a block)<br>3 – performs movement without compensation	Likert 0 to 3 (4 point)

	Modified AAA	As per AAA instructions	CRITERIA:<br>UPPER QUADRANT<br>1 - Perfect hands above head/feet<br>2- Hands above head/feet<br>3 - Unable to achieve position<br>TRIPLE FLEXION<br>1 - Perfect squat to parallel<br>2 - Squat to parallel (compensatory)<br>3 – Unable to achieve position<br><br>HIP CONTROL<br>1 - Neutral spine throughout<br>2 - Loss of control at end of range<br>3 - Excessive deviation	Likert 1 to 3
	Musculoskeletal Readiness Screening Tool	The modified deep squat was initiated with the participant standing barefoot and the shoulders abducted 180 degrees and elbows extended to 90 degrees. With the toes facing directly forward, the subject attempted to squat low enough for the thighs to break parallel with the floor.	CRITERIA:<br>2 – upper torso remained parallel with tibia or vertical, arms in line with torso, femur below horizontal and knees aligned with feet<br>1 – failure to meet one of the above<br>0 - pain	Likert 0 to 2
	Movement Competency Screen	Bodyweight squat<br>Perform a body weight squat with your fingertips on the side of your head and your elbows in line with your ears. Squat as low as you comfortably can at a comfortable speed.	CRITERIA:<br>Head – Held in neutral position<br>Shoulders – Held down and away from ears. Elbows appear in line with ears.<br>Lumbar – Held in neutral curve position.<br>Hips – Horizontally aligned and mobile. Move back and down during flexion.<br>Knees – Aligned with hips and feet during flexion.<br>Ankles – Mobility allows adequate dorsi-flexion during knee and hip flexion.<br>Feet – Stable with heels grounded during lower limb flexion.<br>Balance – Evenly distributed.<br>Depth – Top of thighs appear parallel with floor.	If no then recorded as a point – more points = poorer function
	Movement System Screening Tool	Overhead squat<br>Standing with feet shoulder width apart, toes pointing forward and holding the PVC overhead, he/she squats as deeply as possible 3 times. The rater evaluates from the front, side, and back. If participant was unable to complete the full motion in an error-free fashion, a wooden 2 × 6 board was placed under the heels and the squat was re-evaluated.	CRITERIA<br>0: Pain present during test<br>1: Unable to squat with thighs below parallel without compensations using 2 × 6 board<br>2: Able to squat to below parallel with 2 × 6 board<br>3: Able to squat to below parallel without 2 × 6 board and no compensations.	Likert 0 to 3 (4 points)
	Resistance Training Skills Battery	Provide a demonstration of the movement. Instruct the participant to perform 4 repetitions of the exercise with the arms extended forward at shoulder height. Repeat a second trial.	CRITERIA:<br>1. Feet are shoulder width or slightly wider apart and facing forward<br>2. Back is kept straight and stable throughout the movement<br>3. Knees point in the same direction as feet during movement	Likert 1 to 3 for overall movement<br>3 = high performance (all performed correctly)<br>2 = moderate (most performed correctly)<br>1 = low (few performed correctly)<br><br>Best repetition method used

			4. Heels remain on floor throughout the movement<br>5. Thighs are parallel to the floor at the bottom of the movement	
	Selective Functional Movement Screen	Please start by placing feet shoulder width and toes pointing forward. Please place your arms over your head and move them slightly outside your shoulders with your elbows straight. Hold this position and squat down as far as you can towards the floor	CRITERIA:<br>Loss of shoulder flexion<br>Thoracic Flexes<br>Hips don't break parallel<br>Sagittal plane deviation of lower extremity	Either 1 point for each criteria fulfilled OR<br><br>Categorised as:<br>Functionally non-painful<br>Functional-Painful<br>Dysfunctional-Non Painful<br>Dysfunctional-Painful
One-Legged Squat Test	9+ screening battery	Standing on the left leg with hands in hip position. One legged squat is performed slowly as deep as possible with the upper body vertically. This position should be held briefly before returning to the starting position.	3 points: All criteria have to be fulfilled<br>Hip, knee and foot aligned<br>Pelvis in horizontal line<br>The upper body is vertical<br><br>2 points: One or more of the following criteria<br>Hip, knee and foot aligned<br>Pelvis is not in horizontal line<br>The upper body is not vertical<br><br>1 point: This criterion has to be fulfilled<br>Hip, knee and foot is not aligned	Likert 1 to 3
Single Leg Squat off Box	Athlete Ability Assessment	5 repetitions<br>Athletes were given specific instructions and demonstration on how to correctly perform each movement, including a verbal description of the scoring criteria verbatim from the scoring criteria in Table 1.	CRITERIA<br>Trunk Angle<br>3 – Maintains perfect trunk posture for all reps<br>2 – Inconsistent or uncontrolled forward lean and/or movement from neutral lumbopelvic position<br>1 – Excessive and uncontrolled forward lean and/or movement from neutral lumbopelvic position<br><br>Hip/Knee/Ankle Alignment<br>3 – Perfect alignment and control of hip/knee/ankle throughout every repetition<br>2 – Inconsistent form with some perfect repetitions OR minor misalignment on all repetitions<br>1 – Poor alignment throughout<br><br>Depth<br>3 – Hip below knee (below parallel) while maintaining neutral spine for all reps<br>2 – Depth below parallel for some but not all reps<br>1 – Not able to achieve required depth for any reps	Likert 1 to 3
Single Leg Squat			CRITERIA:<br>Head – Held in neutral position	

	Movement Competency Screen	Cross your arms and place your hands on your shoulders with your elbows pointing straight ahead. Perform a forward lunge then rotate toward the forward knee. Return to centre and then push back to return to the starting position. Alternate with each repetition.	Shoulders – Held down and away from ears. Elbows appear in line with ears.<br>Lumbar – Held in neutral curve position.<br>Hips – Mobile to facilitate flexion and stable to minimize weight shift to over stance leg.<br>Knees – Aligned with hips and feet during flexion.<br>Ankles – Mobility allows adequate dorsi-flexion during knee and hip flexion.<br>Feet – Stable with heels grounded during lower limb flexion.<br>Balance – Maintained on each leg.<br>Depth – Top of thighs appear parallel with floor.	If no then recorded as a point – more points = poorer function
Lunge	9+ screening battery	The athlete is standing in a lunge position. The left knee is aligned with metatarsale II and the knee is touching a vertical pole. The right femur is vertical, right knee joint flexed and the right foot is dorsi-flexed. A measurement is taken between the front and rear digitorum I. A lunge is performed on a 0.1 m plank between those measurements. A pole is held behind the back with the right arm behind the head and the right elbow flexed pointing straight to the side. The left hand is held behind the back at the lumbar spine with a closed fist and the knuckles touching the lumbar spine.	3 points: All criteria have to be fulfilled<br>Pole contact with head and sacrum<br>The upper elbow pointing 90° to the side<br>No movement of upper body with a pole vertical<br>Contact is kept between knuckle and column<br>Both feet in line pointing straight forward<br>The anterior knee is straight over the anterior foot.<br>Anterior heel is kept in the plank<br>Rear foot touch the plank<br><br>2 points: One or more of the following criteria have to be fulfilled<br>No pole contact with head and sacrum<br>The upper elbow not pointing 90° to the side<br>Minor movement of upper body, pole is not vertical<br>No contact between knuckle and columna<br>Feet is not pointing straight forward<br>The anterior knee not in line over foot<br><br>1 point: One or more of the following criteria have to be fulfilled<br>Loss of balance<br>Rear knee is not in contact with the plank<br>The anterior heel is not in contact with the plank.	Likert 1 to 3
	AAA-6	Participants are instructed to place hands on hips.<br><br>1. Lunge forward and backward without stopping.<br>2. Touch your knee on the ground during each repetition.<br>3. Perform 5 repetitions	Deviation from the following criteria were assessed using a 5-point likert scale. The scale was anchored at the following points:<br>1 = very large<br>2 = large<br>3 = moderate<br>4 = small<br>5 = very small<br>6 = criteria fulfilled<br><br>CRITERIA:<br>Single Leg Stability – shoulder, hip, knee, and ankle aligned<br>Take-off control – Controlled movement<br>Trunk control – neutral trunk	Likert 1 to 6

	Athlete Introductory Movement Screen	To standardise delivery and technical instruction across testing sessions, athletes viewed pre-recorded video demonstrations in which the movements were presented and desired movement patterns described using the key points from the scoring criteria (see Table 1). Following the first viewing, athletes were permitted to practise four to five repetitions of the movements and then re-watch the demonstration video.	CRITERIA:<br>Trunk control<br>3 – Maintains neural spine during full movement (out and back), no flexion/extension or rotation for 4 consecutive repetitions<br>2 – 3 appropriate repetitions; OR minor misalignment/slight loss of control on all repetitions<br>1 – 2 or less appropriate repetitions<br><br>Depth<br>3 – Knee of rear leg lowered with control until almost touching the floor (<10 cm) for 4 consecutive repetitions<br>2 – 3 appropriate repetitions; OR near appropriate depth for all repetitions OR some weight acceptance on knee on 1 rep (i.e. visible touch down)<br><br>Frontal plane and repetition<br>3 – Appropriate alignment and control of knee/ankle throughout 4 consecutive repetitions<br>2 – 3 appropriate repetitions; OR minor misalignment on all repetitions<br>1 – 2 or less appropriate repetitions<br><br>Hip/pelvic control<br>3 – Appropriate alignment and control of hips with neutral pelvis throughout movement on 4 consecutive repetitions<br>2 – 3 appropriate repetitions; OR minor misalignment (end range) on all repetitions<br>1 – 2 or less appropriate repetitions	Likert 1 to 3
	Functional Movement Screen<br><br>Modified Functional Movement Screen (1)<br><br>Modified Functional Movement Screen (2)	The tester attains the individual's tibia length, by either measuring it from the floor to the tibial tuberosity or acquiring it from the height of the string during the hurdle step test. The individual is then asked to place the end of their heel on the end of the board or a tape measure taped to the floor. The previous tibial measurement is then applied from the end of the toes of the foot on the board and a mark is made. The dowel is placed behind the back touching the head, thoracic spine, and middle of the buttocks. The hand opposite to the front foot should be the hand grasping the dowel at the cervical spine. The other hand grasps the dowel at the lumbar spine. The individual then steps out on the board or tape measure on the floor placing the heel of the opposite foot at the indicated mark. Both toes must point forward, and feet must begin flat. The individual then lowers the back knee enough to touch the surface behind the heel of the front foot, while maintaining an upright posture, and then returns to the starting position. The lunge is performed up to three times	0 – movement causes pain<br>1 – unable to complete the movement or assume the position required to perform the movement<br>2 – able to complete the movement but must compensate (e.g. dowel loses contact, knee doesn't touch behind foot)<br>3 – performs movement without compensation	Likert 0 to 3 (4 point)

	Lower Extremity Functional Tests	bilaterally in a slow controlled fashion. If one repetition is completed successfully then a three is given for that extremity (right or left).<br><br>From a standing position individuals were instructed to lunge forward (leading with their dominant leg) a distance of approximately one and a half times the length of their normal gait stride. As they moved into single leg stance (on the dominant leg, with the contralateral leg off the ground) they flexed the hip and knee while maintaining an upright trunk. Individuals were instructed to continue the lunge until reaching maximum dorsiflexion of the stance leg without lifting their heel.	Criteria:<br>Trunk – moves out of neutral in frontal or transverse plane<br><br>Pelvis 1 – moves out of neutral in the frontal or transverse plane<br><br>Pelvis 2 – moves away from the midline<br><br>Knee – Patella moves out of line with 2nd toe<br><br>Foot – Moves into excessive pronation<br><br>Oscillation – Observable oscillation (movement to and from neutral)<br><br>Overall movement quality	Scoring:<br>Trunk – No = 0, Yes (minor) = 1, Yes (moderate) = 2, Yes (marked) = 3<br>Pelvis 1 – No = 0, Yes (minor) = 1, Yes (moderate) = 2, Yes (marked) = 3<br>Pelvis 2– No = 0, Yes (minor) = 1, Yes (moderate) = 2, Yes (marked) = 3<br>Knee – No = 0, Yes (minor) = 1, Yes (moderate) = 2, Yes (marked) = 3<br>Foot – No = 0, Yes (minor) = 1, Yes (moderate) = 2, Yes (marked) = 3<br>Oscillation – No = 0, Yes (minor) = 1, Yes (moderate) = 2, Yes (marked) = 3<br><br>Overall movement quality – Acceptable = 0, minor dysfunction = 1, moderate dysfunction = 2, marked dysfunction = 3
	Modified AAA	No description/instructions	CRITERIA:<br>HIP/KNEE/ANKLE<br>1 - Alignment during movement<br>2 - Slight deviation<br>3 - Poor alignment<br><br>HIP CONTROL<br>1 - Neutral hip position<br>2 - Slight deviation<br>3 - Excessive flex/ext<br><br>TAKE-OFF CONTROL<br>1 – Control<br>2 – Jerking<br>3 - Excessive deviation	Likert 1 to 3
Walking Lunge	Athlete Ability Assessment	10 steps<br>Athletes were given specific instructions and demonstration on how to correctly perform each movement, including a verbal description of the scoring criteria verbatim from the scoring criteria.	CRITERIA:<br>Knee/Ankle Alignment<br>3 – Perfect alignment and control of knee/ankle throughout every rep<br>2 – Inconsistent form with some perfect reps OR minor loss of control on all reps	Likert 1 to 3

			1 – Excessive loss of control from neutral throughout the movement<br><br>Hip Control<br>3 – Perfect alignment of hips throughout<br>2 – Inconsistent form with some perfect reps OR minor loss of control on all reps<br>1 – Excessive loss of control from neutral throughout the movement<br><br>Trunk control<br>3 – Maintain neutral spine throughout. No forward or side flexion/movement<br>2 – Inconsistent form with some perfect reps OR minor loss of control on all reps<br>1 – Forced lumbar extension or lack of control during force production	
Lunge and Twist	Movement Competency Screen	Cross your arms and place your hands on your shoulders with your elbows pointing straight ahead. Perform a forward lunge then rotate toward the forward knee. Return to centre and then push back to return to the starting position. Alternate with each repetition.	CRITERIA:<br>Head – Held in neutral position<br>Shoulders – Held down and away from ears. Rotation appears to occur through thoracic spine.<br>Lumbar – Held in neutral curve position. Rotation and/ or lateral flexion does not occur during trunk twisting.<br>Hips – Mobile and stable to prohibit elevation and depression during rotation.<br>Knees – Aligned with hips and feet during flexion and do not move laterally during rotation<br>Ankles – Mobility allows adequate dorsi-flexion during knee and hip flexion.<br>Feet – Heel of lead leg in contact with the floor, trail foot flexed and balanced on forefoot.<br>Balance – Maintained on each leg.<br>Depth – Lead thigh parallel with floor.	If no then recorded as a point – more points = poorer function
Lunge	Resistance Training Skills Battery	Provide a demonstration of the movement with hands on hips. Instruct the participant to perform 4 repetitions on the same leg. Second trial is completed with the other leg.	CRITERIA<br>1. Takes an exaggerated step forward and lands heel first<br>2. Torso is kept upright and stable at all times (no twisting)<br>3. Knee of rear leg is almost touching the floor (approx. 10cm)<br>4. There is alignment between hip, knee and foot of each leg<br>5. Returns to starting position in one movement	Likert 1 to 3 for overall movement<br>3 = high performance (all performed correctly)<br>2 = moderate (most performed correctly)<br>1 = low (few performed correctly)<br><br>Best repetition method used
Active Hip Flexion	9+ screening battery	The athlete is in supine position with extended legs and arms in an anatomic position. A 0.02 m board is placed under the knees. A vertical pole is placed between the Superior anterior iliac spine (SAIS) and mid patellae of the left leg. Left hip is flexed while the right knee remains extended with the feet dorsi-flexed. No rotation of the hip is allowed.	3 points: The following criteria has to be fulfilled<br>Lateral malleol passes the pole with both knees extended and neck in neutral position<br>Both knees extended and neck in neutral position at the mid point of the first given point and mid patellae<br>The right knee is in contact with the plank<br><br>2 points: The following criteria has to be fulfilled	Likert 1 to 3

			Lateral malleol passes the pole between the measurement above and mid patellae<br>Both knees extended and neck in neutral position at half way between first pole position on thigh and SAIS<br>The right knee is in contact with the plank<br><br>1 point: This criterion has to be fulfilled<br>Lateral malleol does not pass the at above given criteria.	
Single Leg Romanian Deadlift	AAA-6	Participants are instructed to place hands on hips<br><br>1. Lower your body in a controlled way to the lowest position possible<br>2. Keep a slight bend in your knee<br>3. Keep your back straight<br>4. Perform 5 repetitions	Deviation from the following criteria were assessed using a 5-point likert scale. The scale was anchored at the following points:<br>1 = very large<br>2 = large<br>3 = moderate<br>4 = small<br>5 = very small<br>6 = criteria fulfilled<br><br>CRITERIA:<br>Trunk control – Neutral trunk position<br>Hip separation – full hip separation<br>Sagittal hip control – no rotation	Likert 1 to 6
	Modified AAA	No description/instructions	CRITERIA:<br>HIP CONTROL – FRONTAL<br>1 – Maintain neutral spine<br>2 – Slight flex/ext through hips<br>3 – Excessive flex/ext on SL stance<br><br>HIP CONTROL – SAGITTAL<br>1 – no rotation<br>2 – slight rotation at end of range<br>3 – Excessive rotation<br><br>HINGE RANGE<br>1 – achieves parallel<br>2 – can dissociate but not reach parallel<br>3 – cannot dissociate hips from trunk	Likert 1 to 3
Straight leg raises	9+ screening battery	Supine with feet and knees together, feet and arms crossed over the chest	3 points: The following criteria has to be fulfilled<br>Ability to stabilize trunk with legs together, dorsi flexed feet with -heels touches the floor and back with retained position of lumbar spine L4-L5 pushed towards the physiotherapist fingers between the floor and the back.<br>Neck in neutral position<br><br>2 points: This criterion has to be fulfilled<br>Ability to stabilize trunk with legs together, as above to 30°<br><br>1 point: This criterion has to be fulfilled<br>No ability to stabilize trunk with 30° hip flexion.	Likert 1 to 3

Push up	9+ screening battery	Prone with forehead on the floor with extended knees, feet together and hands shoulder width apart. Perform a push-up with the body as a unit and a stick placed on the back with a pincett grip at the lumbar spine to control that the lumbar spine is stable.	3 points: Both criteria have to be fulfilled<br>The body is pushed up as a unit facing straight down through the whole motion<br>Contact is kept between pole, back of the head as well as between the testers fingers and lumbar spine<br><br>2 points: The following criteria have to be fulfilled<br>The body is pushed up as a unit facing straight down through the whole motion<br>Contact is not kept between stick and back of the head or between the PTs fingers and lumbar spine<br><br>1 point: This criterion has to be fulfilled The body is not pushed up as a unit	Likert 1 to 3
	AAA-6	To standardize hand placement, participants are instructed to place their hands just outside shoulder width (thumbs in line with most lateral aspect of the shoulder).<br><br>1. In a controlled fashion, lower your chest to the mat and push up until elbows are fully extended.<br>2. Perform 20 (junior participants) or 30 (senior participants) repetitions.	Deviation from the following criteria were assessed using a 5-point likert scale. The scale was anchored at the following points:<br>1 = very large<br>2 = large<br>3 = moderate<br>4 = small<br>5 = very small<br>6 = criteria fulfilled<br><br>CRITERIA:<br>Total body control – shoulder-heel aligned<br>Scapula control – stable and move symmetrically<br>Repetitions – 1 = 1-9, 3 = 10-19, 5 = 20-29, 6 = 30	Likert 1 to 6
	Athlete Ability Assessment	20 minimum reps (males)<br>12 minimum reps (females)<br>Athletes were given specific instructions and demonstration on how to correctly perform each movement, including a verbal description of the scoring criteria verbatim from the scoring criteria.	CRITERIA:<br>Scapulohumeral rhythm<br>3 – Scapula depression and retraction constant throughout the movement. No protraction or elevation of scapular or flaring of elbows.<br>2 – Inconsistent form. Some perfect repetitions.<br>1 – Poor scapula positioning and control for all reps.<br><br>Body control<br>3 – Perfect body control and alignment for every repetition.<br>2 – Perfect body control and alignment for some but not all reps<br>1 – Poor body control and/or alignment for all reps<br><br>Complete reps<br>3 – M > 20, F > 12<br>2 –<br>1 – M < 20, F < 6	Likert 1 to 3
	Athlete Introductory Movement Screen	To standardise delivery and technical instruction across testing sessions, athletes viewed pre-recorded video	CRITERIA<br>Upper body alignment/control	Likert 1 to 3

		demonstrations in which the movements were presented and desired movement patterns described using the key points from the scoring criteria (see Table 1). Following the first viewing, athletes were permitted to practise four to five repetitions of the movements and then re-watch the demonstration video	3 – Head, back and hips are held in a straight line throughout the movement on 4 consecutive repetitions.<br>2 – 3 appropriate repetitions, OR minor misalignment and/or asymmetry on all repetitions<br>1 – 2 or less appropriate repetitions<br><br>Shoulder position and control<br>3 – Shoulders are away from ears (not shrugged/moved closer to ears during movement) AND elbow positioning is directed slightly anterior, not flaring, in 4 consecutive repetitions<br>2 – 3 appropriate repetitions; OR minor misalignment/slight loss of control on all repetitions<br>1 – 2 or less appropriate repetitions<br><br>Hand position<br>3 – Hands are placed under the shoulder AND hands not repositioned in any repetitions<br>2 – Minor mis-positioning of hands relative to shoulders (less than 10 cm); OR 1 repositioning of hands<br>1 – Poor initial positioning of hands OR 2+ repositioning of hands	
	Modified AAA	As per AAA instructions	CRITERIA:<br>TRUNK/BODY CONTROL<br>1 – Perfect control/alignment<br>2 – Perfect control for some<br>3 – Poor body control for all<br><br>UPPER QUADRANT<br>1 – Perfect form/symmetry<br>2 – Inconsistent<br>3 – Poor scap. positioning for all reps<br><br>X 30 REPS<br>1 – Hits target counts (30)<br>2 –<br>3 - < 10 reps	Likert 1 to 3
	Movement Competency Screen	Perform a standard push up.	CRITERIA:<br>Head – Held in neutral position appears centrally aligned<br>Shoulders - Held down and away from ears. Scapulae movement balanced and rhythmic and not excessively abducted during arm extension.<br>Lumbar – Held in neutral position<br>Hips - Held in line with the body during arm flexion and extension.<br>Knees - Extended.<br>Ankles - NR.<br>Feet - Feet straight, heels not falling in or out.<br>Balance - NR.<br>Depth – Chest touches floor	If no then recorded as a point – more points = poorer function

Trunk stability push up	Functional Movement Screen<br><br><br>Modified Functional Movement Screen (1)<br><br>Modified Functional Movement Screen (2)	The individual assumes a prone position with the feet together. The hands are placed shoulder width apart at the appropriate position per the described criteria. During this test, men and women have different starting arm positions. Men begin with their thumbs at the top of the forehead, while women begin with their thumbs at chin level. The knees are fully extended and the ankles dorsiflexed. The individual is asked to perform one push up in this position. The body should be lifted as a unit; no "lag" (or arch) should occur in the lumbar spine when performing the movement. If the individual cannot perform a push-up in this position, the thumbs are moved to the next easiest position, chin level for males, shoulder level for females, and the push-up is attempted again. The trunk stability push up can be performed a maximum of three times.	0 – pain during movement or positive spinal extension clearing exam<br>1 – lag in torso and chest rising<br>2 – regression in hand position<br>3 – clearance in male/female hand position	Likert 0 to 3 (4 point)
Push Up	Resistance Training Skills Battery	Provide demonstrations of modified (on knees) and full (on toes) push-ups. Instruct the participant to perform 4 modified or full push-ups. Repeat a second trial.	CRITERIA:<br>1. Hands are shoulder width or slightly wider apart<br>2. Head, back and hips are held in a straight line throughout the movement<br>3. Body is lowered until elbows are at a 90 degree angle<br>4. Shoulders are held down and away from ears (shoulders are not shrugged)	Likert 1 to 3 for overall movement<br>3 = high performance (all performed correctly)<br>2 = moderate (most performed correctly)<br>1 = low (few performed correctly)<br><br>Best repetition method used
Diagonal Lift (Bird-Dog)	9+ screening battery	Starting position in quadruped with shoulders and hips at 90° relative to the torso, with the right hand and left foot and knee placed on a line. The knees are positioned at 90° and the ankles are plantar-flexed. The athlete then flexes the right shoulder and extends the left hip and knee. Then the left shoulder is extended and the right knee is flexed enough for the elbow and knee to touch under the stomach	3 points: All of the following criteria have to be fulfilled:<br>Performs one diagonal lift with the right hand and the opposite foot and knee on a line No visible rotation in the spine<br>Fully extended leg and arm in the horizontal plane<br>No abduction in either leg or arm<br>No winging of the scapula<br><br>2 points: All of the following criteria have to be fulfilled<br>Performs one diagonal lift with hand and the opposite knee on each side of a line<br>No visible rotation in the spine<br>Fully extended leg and arm in the horizontal plane<br>No abduction in either leg or arm<br>No winging of the scapula<br><br>1 point: One or more of the following criteria have to be fulfilled<br>Performs one diagonal lift with hand and the opposite knee on each side of a line, with one or more of the following compensatory movement pattern<br>Visible rotation in the spine<br>Not fully extended leg and arm in the horizontal plane<br>Abduction in either leg or arm Winging of the scapula].	Likert 1 to 3

Seated Rotation	9+ screening battery	Starting position is sitting on the floor on both tuber ischii with the legs crossed and with the spine as straight as possible holding a pole in front of the chest. A second pole, held by the physiotherapist is positioned vertically between the feet. The athlete is asked to retract their scapulas so the pole is in touch with the chest and thereafter slowly rotate to the left.	3 points: This criterion has to be fulfilled.<br>Performs a slow rotation with the pole in touch with the chest until the poles touch each other.<br><br>2 points: This criterion has to be fulfilled<br>Performs a slow rotation with the pole in touch with the chest more then 45°. The poles are not touching each other.<br><br>1 point: This criterion has to be fulfilled Performs a slow rotation with the pole in touch with the chest less then 45°	Likert 1 to 3
Shoulder Mobility	9+ screening battery	Starting position is standing with feet shoulder width apart. Measure the length distance between the distal wrist crease to the tip of the third digit. The athlete is instructed to make a fist with each hand, placing the thumbs inside the fists and placing them as close as possible together behind the back. The tester measures the distance between the two fists	3 points: This criterion has to be fulfilled Less then one hand length between the fists<br><br>2 points: This criterion has to be fulfilled Less then one and a half hand length between the fists<br><br>1 point: This criterion has to be fulfilled One and a half hand length or more between the fists].	Likert 1 to 3
	Basic Functional Assessment Protocol	-Spread your arms and keep them horizontally (in cross) and place your thumbs inside the other fingers.<br>-When I say "Ready, go" lift your right arm above your head and bring the left arm below until both wrists come together in your back.<br>-You will perform two repetitions for each side.	VIEW: BACK<br>Peak scapula, internal rotation arm<br>- Pronunciation of the lower scapular peak when the right/left arm is in internal/external rotation simultaneously<br><br>VIEW: SAGITTAL<br>Excess lumbar lordosis, internal rotation arm<br>- The lumbar curve concavity increases too much during movement when the left/right arms is in internal/external rotation simultaneously<br>Cervical protraction, internal rotation arm<br>The pterigoidea vertical line is forwarded to an earlier position when the right/left arm is in internal/external rotation simultaneously	If "Yes" to the criteria then 1 point, maximum points is better function
Reciprocal Shoulder Mobility	Arm Care Screen	The participant began in standing with feet together and both hands open. The participant simultaneously reached one hand behind their head and other hand behind and up their back, similar to an Apley's Scratch test position, assuming an extended and internally rotated position with one shoulder and a flexed and externally rotated position with the other. The arms must move in one smooth motion and tall posture must be maintained while the participant attempts to touch the fingertips of both hands together.	Inability to touch right and left fingertips together on both reach directions was considered a failure in the test.	Pass/Fail
Shoulder Mobility	Functional Movement Screen	The tester first determines the hand length by measuring the distance from the distal wrist crease to the tip of the third digit in inches. The individual is then instructed to	0 – pain during movement<br>1 – fists are not within 1.5 fist length	Likert 0 to 3 (4 point Likert)

		make a fist with each hand, placing the thumb inside of the fist. They are then asked to assume a maximally adducted, extended, and internally rotated position with on shoulder and a maximally abducted, flexed, and externally rotated position with the other. During the test, the hands should remain in a fist and the fists should be place on the back in one smooth motion. The tester then measures the distance between the two closest bony prominences.	2 – fists are within 1.5 fists length<br>3 - fists are within 1 fist's length	
	Movement System Screening Tool	In one motion while standing, participant moves the right fist overhead and down the back as far as possible while simultaneously taking the left fist up the back as far as possible. Do not ''creep'' the hands closer after initial placement. Measure the distance between the two closest bony prominences on the hands (measured to nearest 0.5 in.. Measure hand length prior to testing (most distal wrist crease to the tip of the third digit). Raters record the distance of the closest reach. If the participant can touch the hands together, then a distance of zero is recorded for that side.	CRITERIA:<br>0: Pain present during test<br>1: >1.5 hand lengths apart<br>2: > 1 hand length apart<br>3: < 1 hand length apart<br>Sides scored separately.	Likert 0 to 3 (4 point Likert)
Shoulder clearing test	Functional Movement Screen	The individual places his/her hand on the opposite shoulder and then attempts to point the elbow upward	Positive response = 0 on shoulder mobility	Pass/Fail
Chin Up	AAA-6	To standardize hand placement, participants are instructed to place their hands just outside shoulder width (thumbs in line with most lateral aspect of the shoulder).<br><br>1. Pull yourself up until your chin is above the bar.<br>2. Lower yourself until your elbows are straight.<br>3. Perform 10 repetitions.	Deviation from the following criteria were assessed using a 5-point likert scale. The scale was anchored at the following points:<br>1 = very large<br>2 = large<br>3 = moderate<br>4 = small<br>5 = very small<br>6 = criteria fulfilled<br><br>CRITERIA:<br>Total body control – shoulder-knee aligned<br>Scapula control – stable and move symmetrically<br>Repetitions – 1 = 1-3, 3 = 4-6, 5 = 7-9, 6 = 10	Likert 1 to 6
	Athlete Ability Assessment	Minimum reps = 10 (M), 4 (F)<br>Athletes were given specific instructions and demonstration on how to correctly perform each movement, including a verbal description of the scoring criteria verbatim from the scoring criteria.	CRITERIA:<br>Scapulohumeral rhythm<br>3 – Scapula depression and retracted throughout hang. Symmetry of scapulohumeral rhythm during pull and lowering phase of exercise. No scapula elevation or winging.<br>2 – Inconsistent form. Some perfect repetitions OR slight asymmetry<br>1 – Poor scapula positioning and control for all repetitions.	Likert 1 to 3

	Modified AAA	As per AAA instructions	Body control<br>3 – No swinging. Perfect body control for all repetitions<br>2 – Perfect body control for some repetitions<br>1 – Poor scapula positioning and control for all repetitions<br><br>Complete repetitions<br>3 – M > 10, F < 4<br>2 –<br>1 – M < 10, F > 4<br><br>CRITERIA:<br>SCAPULA RHYTHM<br>1 – Perfect form/symmetry<br>2 – Inconsistent, some perfect<br>3 – Unable to achieve position<br><br>TOTAL BODY CONTROL<br>1 – Perfect control/alignment<br>2 – Perfect control/alignment for some<br>3 – Poor body control for all reps<br><br>X 10 REPS<br>1 – Hits target counts<br>2 –<br>3 - < 10 reps	Likert 1 to 3<br><br>Can score 2 as a "3 with 1 elsewhere" – Rogers et al., 2019
90/90 Total Body Rotation	Arm Care Screen	The participant assumes a standing position with feet together, toes pointing forward and arms in the 90/90 position (90° shoulder abduction and 90° elbow flexion). The participant rotates their entire body including the hips, shoulders, and head as far as possible to the right while the foot position remained unchanged.	Inability to see the back shoulder when viewing the participant from behind on both sides was considered failure on the test.	Pass/Fail
Lower Body Diagonal Reach<br><br>(Posteromedial Reach)	Arm Care Screen	The participant stood two shoe lengths away from a wall and while maintaining single leg balance on one foot the participant reaches with the opposite foot behind and across their body to try to touch the point on the wall just above the ground five consecutive times without the foot touching down or losing balance.	Inability to touch the wall five consecutive times or loss of balance on either side was considered failure on the test.	Pass/Fail
Prone Hold on Hands	Athlete Ability Assessment	2 min<br>Athletes were given specific instructions and demonstration on how to correctly perform each movement, including a verbal description of the scoring criteria verbatim from the scoring criteria.	CRITERIA<br>Upper back/shoulder position<br>3 – Scapula depression and retraction constant for 2 min. No protraction or elevation<br>2 – Inconsistent positioning (repositioning) throughout the 2 min<br>1 – Unable to attain correct position<br><br>Hip position<br>3 – Neutral hip positioning with no anterior/posterior tilt or rotation<br>2 – Inconsistent positioning (repositioning) throughout the 2 min	Likert 1 to 3

			1 – Unable to attain correct position<br><br>Time<br>3 - > 2 min<br>2 – 1-2 min<br>1 - < 1 min	
Front support brace with shoulder touches	Athlete Introductory Movement Screen	To standardise delivery and technical instruction across testing sessions, athletes viewed pre-recorded video demonstrations in which the movements were presented and desired movement patterns described using the key points from the scoring criteria (see Table 1). Following the first viewing, athletes were permitted to practise four to five repetitions of the movements and then re-watch the demonstration video.	CRITERIA<br>Foot contact<br>3 – Both feet remain on the ground throughout 4 consecutive repetitions, no foot sliding<br>2 – 3 appropriate repetitions (1 foot lift only); OR no repetitions lifted, but feet slide on up to 2 repetitions<br>1 – 2 or less appropriate repetitions (feet lifted in 2 or move AND/OR feet slide in 3 or more)<br><br>Full body alignment<br>3 – Holds the total body plane in straight alignment through legs (knees fully extended), hips, shoulders and head, for 4 consecutive repetitions<br>2 – 3 appropriate repetitions; OR minor misalignment on all repetitions<br>1 – 2 or less appropriate repetitions<br><br>Resists rotation<br>3 – Minimal rotation of the pelvis/hip complex during all 8 repetitions while changing from 4 to 3 points of contact (approx. 10 cm is acceptable)<br>2 – 6 or more appropriate repetitions; OR minor rotation on all repetitions<br>1 – 6 or less appropriate repetitions<br><br>Controlled arm movements<br>3 – Chest touches performed in controlled manner with arms deliberately returned to the floor following chest touch on all 8 repetitions<br>2 – 6 or more appropriate repetitions; OR minor loss of control on all repetitions<br>1 – 6 or less appropriate repetitions	Likert 1 to 3
	Resistance Skills Training Battery	Provide a demonstration of the movement. Instruct the participant to perform 2 repetitions per side alternating sides each repetition. Repeat a second trial.	CRITERIA:<br>1. Straight line through legs, hips, shoulders and head<br>2. Feet are approximately shoulder width apart<br>3. Minimal rotation of body while changing hand placement (approx. 10cm is acceptable)<br>4. Both feet remain on the ground throughout the entire trial<br>5. Chest touches are performed in a controlled manner	Likert 1 to 3 for overall movement<br>3 = high performance (all performed correctly)<br>2 = moderate (most performed correctly)<br>1 = low (few performed correctly)<br><br>Best repetition method used

Lateral Hold on Hands	Athlete Ability Assessment	2 min – Left and Right Sides<br>Athletes were given specific instructions and demonstration on how to correctly perform each movement, including a verbal description of the scoring criteria verbatim from the scoring criteria.	CRITERIA<br>Upper back/shoulder position<br>3 – Scapula depression and retraction constant for 2 min. No protraction or elevation<br>2 – Inconsistent positioning (repositioning) throughout the 2 min<br>1 – Unable to attain correct position<br><br>Hip position<br>3 – Neutral hip positioning with no anterior/posterior tilt or rotation<br>2 – Inconsistent positioning (repositioning) throughout the 2 min<br>1 – Unable to attain correct position<br><br>Time<br>3 - > 2 min<br>2 – 1-2 min<br>1 - < 1 min	Likert 1 to 3
Side bridge with active hip abduction	Movement system screening tool	Participant lies on his/her side, propped up on the forearm with the shoulder over the elbow, and the bottom knee bent to 90◦. Top and bottom thighs should be in line with one another. Top leg should be straight, toes lifted towards the shin and pointed forward. Participant lifts pelvis off the floor/table until head, spine, and bottom leg are in a straight line, then raises the top leg.	CRITERIA<br>0: Pain present during test<br>1: Obvious deviations were noted<br>2: Subtle deviations were noted<br>3: No compensations or deviations were present<br>Deviations:<br>- Pelvis or spine did not remain in a neutral position<br>- Lack of symmetry in performance between sides<br>- Any trunk movement Sides scored separately	Likert 0 to 4
Single Leg Forward Hop	Athlete Ability Assessment	3 repetitions – Left and Right Sides<br>Athletes were given specific instructions and demonstration on how to correctly perform each movement, including a verbal description of the scoring criteria verbatim from the scoring criteria.	Hip/Knee/Ankle Alignment<br>3 – Perfect alignment of hip/knee/ankle<br>2 – Inconsistent form with some perfect reps OR minor misalignment on all reps<br>1 – Poor alignment throughout<br><br>Balance/Control<br>3 – Landing with perfect balance and control<br>2 – Sticks landing but is unbalanced. Adjustments made via other body movements<br>1 – No balance/control on landing<br><br>Power position on Landing<br>3 – Lands in Single Leg power position/quarter squat after every rep<br>2 – Inability to land in power position on some but not all reps OR makes adjustments post-landing to attain power position<br>1 – Excessive hip/knee/ankle flexion. Poor positioning to reproduce force.	Likert 1 to 3
Hop Lunge	Lower Extremity Functional Tests	From a standing position individuals were instructed to jump forward a distance of approximately 1.0 m and on	Criteria:<br>Trunk – moves out of neutral in frontal or transverse plane	Scoring:

		landing on the dominant leg to flex the hip and knee. Individuals were instructed to continue the lunge until reaching maximum dorsiflexion of the dominant leg without lifting their heel.	Pelvis 1 – moves out of neutral in the frontal or transverse plane<br><br>Pelvis 2 – moves away from the midline<br><br>Knee – Patella moves out of line with 2nd toe<br><br>Foot – Moves into excessive pronation<br><br>Oscillation – Observable oscillation (movement to and from neutral)<br><br>Overall movement quality	Trunk – No = 0, Yes (minor) = 1, Yes (moderate) = 2, Yes (marked) = 3<br>Pelvis 1 – No = 0, Yes (minor) = 1, Yes (moderate) = 2, Yes (marked) = 3<br>Pelvis 2– No = 0, Yes (minor) = 1, Yes (moderate) = 2, Yes (marked) = 3<br>Knee – No = 0, Yes (minor) = 1, Yes (moderate) = 2, Yes (marked) = 3<br>Foot – No = 0, Yes (minor) = 1, Yes (moderate) = 2, Yes (marked) = 3<br>Oscillation – No = 0, Yes (minor) = 1, Yes (moderate) = 2, Yes (marked) = 3<br><br>Overall movement quality – Acceptable = 0, minor dysfunction = 1, moderate dysfunction = 2, marked dysfunction = 3
Tuck Jump	Tuck Jump Assessment<br><br><br><br><br><br><br><br><br><br><br><br><br><br><br>Musculoskeletal Readiness Screening Tool	10 repetitions<br>Athletes start in the athletic position with feet shoulder-width apart. They initiate the jump with a slight crouch downward while extending their arms behind them. They then swing their arms forward as they simultaneously jump straight up and pull their knees up as high as possible. At the highest point of the jump, the athletes are in the air with thighs parallel to the ground. When landing, the athletes should immediately begin the next tuck jump. Encourage the athletes to land softly, using a toe to mid-foot rocker landing. The athletes should not continue this jump if they cannot control the high landing force or if they demonstrate a knock-kneed landing.<br><br><br>The subject initiated a jump with arms extended behind the subject and while swinging the arms forward the subject jumped vertically, pulled the knees up as high as possible and then attempted to land softly in the same position. This was repeated quickly three times such that each jump occurred immediately upon landing from the preceding jump. If the subject could perform three jumps with thighs at least oriented 45 degrees in the sagittal plane about a coronal axis, landing in approximately the same position with a soft landing 2 points were awarded.	CRITERIA:<br>Knee and thigh motion<br>1. Lower extremity valgus on landing<br>2. Thighs do not reach parallel (peak of jump)<br>3. Thighs not equal side to side (during flight)<br>Foot position during landing<br>4. Foot placement not shoulder width apart<br>5. Foot placement not parallel (front to back)<br>6. Foot contact timing not equal<br>7. Excessive landing contact noise<br>Plyometric Technique<br>8. Pause between jumps<br>9. Technique declines prior to 10 seconds<br>10. Does not land in same footprint (excessive in flight motion)<br><br>CRITERIA:<br>2 – soft landing, 3 in a row with thighs at least 45 degrees landing in the same position<br>1 – one of the above criteria broken<br>0 - painful	Pass/Fail<br><br><br><br><br><br><br><br><br><br><br><br><br><br><br>Likert 0 to 2

Hurdle Step	Basic Functional Assessment Protocol	- Climb on a step (10–20 cm high) and put your bare feet together.<br>- Cross your arms over your chest.<br>- When I say "ready, go" lift your right/left knee as much as you can without falling and keep it up until I tell you.<br>- You will perform two repetitions for each side.	VIEW: FRONT<br>External rotation support foot<br>- Turning the foot on the longitudinal axis until the phalanges of the second right/left toe are oriented in a lateral direction<br>Internal rotation support foot<br>- Turning the foot on the longitudinal axis until the phalanges of the second right/left toe are oriented in the medial direction<br>Knee valgus support<br>- Displacement of the right/left knee in the front plane during movement by staying closer to the midline of the body at the end of the movement<br>Knee varus support<br>- Displacement of the right/left knee in the front plane during movement farther to the midline of the body at the end of the movement.<br>Hip rotation external flexion<br>- Hip rotation in right/left flexion on the longitudinal axis, leaving the leg in bending orientation out of the medial line of the body<br>Internal rotation hip flexed<br>- Hip rotation in right/left flexion on the longitudinal axis, leaving the leg in flexion oriented to the medial line of the body<br>Pelvis tilt<br>- Opposite pelvis drops in the front plane relative to right/left hip flexion<br>Pelvis rotation<br>- Rotation of the hip staying aside, more moved forward than other<br>Thorax rotation towards the hip in flexion<br>- Rotation of the thorax toward the right/left support leg<br>Thorax rotation opposite hip in flexion<br>- Rotation of the thorax in the opposite direction to the right/left support leg<br>Thorax movement<br>- Movement of the thorax in several directions when flexing the right/left hip<br><br>VIEW: BACK<br>Support front pronation<br>- A fall of the plantar arch is observed in the right/left support midfoot area<br>Support foot supination<br>- The plantar arch of the right/left midfoot support is excessively pronounced<br><br>VIEW: SAGITTAL<br>Heels lift, support foot	If "Yes" then 1 point, maximum points = maximum function

			- The heel of the right/left foot loses contact with the support surface<br>Lumbo-pelvis dissociation loss, hip in flexion<br>- Lumbar neutral curvature disappears when flexing the right/left hip<br>Excess lumbar lordosis<br>- The concavity of the lumbar curve during movement when flexing the right/left hip<br>Excess thoracic kyphosis, hip in flexion<br>- The concavity of the lumbar curve is lost excessively during movement, the lumbar curve is rounded by flexing the right/left hip	
	Functional Movement Screen<br><br>Modified Functional Movement Screen (1)<br><br>Modified Functional Movement Screen (2)	The individual assumes the starting position by first placing the feet together and aligning the toes touching the base of the hurdle. The hurdle is then adjusted to the height of the athlete's tibial tuberosity. The dowel is grasped with both hands and positioned behind the neck and across the shoulders. The individual is then asked to maintain an upright posture and step over the hurdle, raising the foot toward the shin, and maintaining alignment between the foot, knee, and hip, and touch their heel to the floor (without accepting weight) while maintaining the stance leg in an extended position. The moving leg is then returned to the starting position. The hurdle step should be performed slowly and as many as three times bilaterally	0 – movement causes pain<br>1 – unable to complete the movement or assume the position required to perform the movement<br>2 – able to complete the movement but must compensate (e.g. flex forward at trunk, ER at hip)<br>3 – performs movement without compensation	Likert 0 to 3 (4 point)
	Movement System Screening Tool	Participant stands with feet together and toes touching the board. Grasping the dowel with both hands, he/she places the dowel behind his/her neck and across the shoulders. From this position, the participant maintains an upright posture, raises the right leg and step over the hurdle, making sure to keep the moving foot toes towards the shin. Once hurdle is cleared, participant touches the floor with his/her heel and returns to the starting position. Alignment of the ankle, knee and hip should be maintained.	CRITERIA:<br>0: Pain present during test<br>1: Contact between the moving foot and hurdle or loss of balance was observed<br>2: Alignment was lost between hips, knees and ankles or the dowel did not remain horizontal<br>3: Test was performed without any compensation<br>*Leg stepping over the hurdle is the leg being scored<br>Sides scored separately.	Likert 0 to 3 (4 point)
Forward step-down test	Basic Functional Assessment Protocol	- Climb on a step (10–20 cm high) and put your bare feet together<br>- Cross your arms over your chest<br>- When I say "ready, go" carry your right/left heel down and forward as much as you can without falling<br>- You will perform two repetitions for each side	VIEW: FRONT<br>External rotation support foot<br>- Turning the foot on the longitudinal axis until the phalanges of the second right/left toe are oriented in a lateral direction<br>Internal rotation support foot<br>- Turning the foot on the longitudinal axis until the phalanges of the second right/left toe are oriented in the medial direction<br>Knee valgus	If "Yes" then 1 point, maximum points = maximum function

			- Displacement of the right/left knee in the front plane during movement by staying closer to the midline of the body at the end of the movement<br>Knee varus support<br>- Displacement of the right/left knee in the front plane during movement, farther to the midline of the body at the end of the movement<br>Extended rotation extended leg<br>- Right/left extended leg rotation on the longitudinal axis, leaving the extended leg facing out of the medial line of the body<br>Internal rotation extended leg<br>- Right/left extended leg rotation on the longitudinal axis, leaving the extended leg facing into the medial line of the body<br>Pelvis tilt<br>- Pelvis drop in the front plane to the side of the leg in right/left extension<br>Pelvis rotation<br>- Rotation of the hip staying aside, more moved forward to other<br>Thorax rotation towards the leg supported<br>- Rotation of the thorax towards the right/left support leg<br>Thorax rotation opposite the leg supported<br>- Rotation of the thorax opposite direction the right/left support leg<br>Thorax movement<br>- Movement of the thorax in several directions when lengthening right/left leg<br><br>VIEW: BACK<br>Support foot pronation<br>- A fall of the planar arch is observed in the right/left support midfoot area<br>Support foot supination<br>- The planar arch of the right/left midfoot support is excessively pronounced<br><br>VIEW: SAGITTAL<br>Heels lift, support foot<br>- The heel of the right/left foot loses contact with the support surface<br>Lumbo-pelvis dissociation loss, the leg support<br>- Lumbar neutral curvature disappears when lengthening right/left leg<br>Excess lumbar lordosis, the leg supported<br>- The concavity of the lumbar curve increases excessively during movement when lengthening right/left leg<br>Excess thoracic kyphosis, the leg supported	

			- The concavity of the lumbar curve is lost excessively during movement, the lumbar curve is rounded when lengthening right/left leg	
	Musculoskeletal Readiness Screening Tool	The forward step down with eyes closed began with the shod subject standing on a standard 8-inch step with the feet approximately shoulder width apart. The subject held two hardcover textbooks weighing approximately 6.8 kilograms at navel level with elbows flexed to 90 degrees and eyes closed. The textbooks were not permitted to make contact with the subject anywhere other than the hands. The subject stepped down with one leg at a time while the investigator stood in front of the subject for safety	CRITERIA:<br>2 – kept eyes closed and no deviation of lower extremeties in the frontal plane<br>1 – eyes opened, loud foot landing or any frontal plane deviation<br>0 – painful	Likert 0 to 2
	Movement System Screening Tool	Participant stands on a 20-cm stool, crosses his/her arms across the chest, and squats down as far as possible (attempting to touch the reaching heel to the ground) five times consecutively. The movement is performed in a slow and controlled manner, while maintaining balance. If unable to touch the reaching heel to the ground, he/she is instructed to stop at approximately 60◦ of knee flexion. Raters provide verbal feedback, if needed, to those unable to reach the heel to the ground. This ensures that any deviations are not a function of step height.	CRITERIA:<br>0: Pain present during test<br>1: Obvious deviations were noted<br>2: Subtle deviations were noted<br>3: No compensations or deviations were present<br>Deviations:<br>- Pelvis and trunk did not remain neutral<br>- Knee collapsed toward midline of the body<br>- Stance heel lifted from the step or loss of balance<br>- Inability to achieve at least 60◦ knee flexion<br>Sides scored separately	Likert 0 to 3 (4 point scale)
	Step Down Test	1. The subject is asked to stand in a single limb support with the hands on the waist, the knee straight and the foot positioned close to the edge of the 20cm box<br>2. The contralateral leg is positioned over the floor adjacent to the step and is maintained with the knee in extension<br>3. The subject then bends the testing knee until the contralateral leg gently touches the floor and then re-extends the knee to the start position<br>4. The manoeuvre is repeated 5 times	CRITERIA:<br>1. Arm strategy – If subject used an arm strategy in an attempt to recover balance add a point<br>2. Trunk movement – If the trunk leaned to one side, add a point<br>3. Pelvis position – If pelvis is rotated or elevated on one side compared to the other, one point is added<br>4. Knee position - If the knee deviated medially and the tibial tuberosity crossed an imaginary vertical line over the second toe, add one point. If the tibial tuberosity crossed the medial boundary of the foot, add two points<br>5. Maintain steady unilateral stance – If the subject stepped down on the non-tested side, or if the subject's tested limb became unsteady (wavered from side to side), add one point	Pass/fail – add points according to criteria, greater points = greater dysfunction

Active Straight Leg Raise	Basic Functional Assessment Protocol	- Lie on your back with your legs extended and your arms stood on the floor a bit separated from the body.<br>- When I say "ready, go" lift your right leg fully extended as much as you can<br>- You will perform two repetitions for each side	VIEW: SAGITTAL<br>External hip rotation, extended hip<br>- Turn the right/left supported leg on the longitudinal axis until the phalanges of the second toe are oriented in a lateral direction<br><br>Internal rotation, extended hip<br>- Turn the right/left supported leg on the longitudinal axis until the phalanges of the second toe are oriented in a medial direction<br><br>Extended leg modification<br>- Modification of the starting posture on the support leg right/left along the movement<br><br>Modification of the raised leg<br>- Flexing the knee right/left leg when it is raised with the hip in flexion<br><br>Flexion thoracic, hip flexion<br>- The thoracic area inclines, keeping the thorax faced in caudal sense when the right/left leg rises<br><br>Extension thoracic, hip flexion<br>- The thoracic area extends, keeping the thorax faced in the cranial sense when the right/left leg is raised<br><br>Extension lumbar, thoracic, hip flexion<br>- The lumbar curve concavity increases too much when the right/left leg is raised<br><br>Flexion lumbar thoracic, hip flexion<br>- The lumbar curve concavity disappears completely when the right/left leg is raised<br><br>Extension cervical, thoracic, hip flexion<br>- The plane of Frankfurt is inclined, leaving the face oriented in a cranial direction when the right/left leg rises<br><br>Flexion cervical, thoracic, hip flexion<br>- The plane of Frankfurt is inclined, leaving the face oriented in a flow direction when the right/left leg rises	If "Yes" then 1 point, maximum points = maximum function
	Functional Movement Screen<br><br><br>Modified Functional Movement Screen (2)	The individual first assumes the starting position by lying supine with the arms in anatomical position, legs over the 2 x 6 board, and head flat on the floor. The tester then identifies the midpoint between the anterior superior iliac spine, and the midpoint of the patella of the leg on the floor, and a dowel is placed at this position, perpendicular to the ground. Next the individual is instructed to slowly lift the test leg with a	0 - pain during movement<br>1 – malleolus below the knee joint<br>2 – malleolus between the knee joint and mid thigh<br>3 – malleolus between mid thigh and ASIS	Likert 0 to 3 (4 point)

		dorsiflexed ankle and an extended knee. During the test the opposite knee (the down leg) must remain in contact with the ground and the toes pointed upward, and the head in contact with the floor. Once the end range position is achieved, note the position of the upward ankle relative to the non-moving limb. If the malleolus does not pass the dowel, move the dowel, much like a pumb line, to equal with the malleolus of the test leg, and score per the criteria.		
	Movement System Screening Tool	Participant lies supine on a mat table with a 2 × 6 board directly under the knees, toes pulled towards the shin, and the arms by the side. From this position, participant raises the one leg as high as possible, while keeping the raised leg straight and the opposite knee against the board.	CRITERIA:<br>0: Pain present during test<br>1: Vertical line of the malleolus of the moving leg is below the knee joint line of the non-moving leg<br>2: Vertical line of malleolus of moving leg is between the mid-thigh and knee joint line of the non-moving leg<br>3: Vertical line of the malleolus of the moving leg is above the mid-thigh of the non-moving leg<br>Sides scored separately	Likert 0 to 3 (4 points)
Closed Kinetic Chain Upper Body Extremity Stability Test	Closed Kinetic Chain Upper Body Extremity Stability Test	Assume a push up position with hands 92 cm apart. In 15 seconds complete as many touches as possible of the opposite hand in an alternating fashion. There are 3 attempts and the greatest number of touches in the 3 attempts is recorded as a score.	Touch the opposite hand with body remaining in a push up position.	Number of touches.
Modified CKCUEST	Modified CKCUEST (Taylor et al., 2016)	Assume push up position with hands under your shoulders.	Touch the opposite hand with body remaining in a push up position.	Number of touches
CKCUEST	Musculoskeletal Readiness Screening Tool	Males started in the push-up position and females began in the kneeling push-up position. With the back slightly inclined in relation to the floor and the hands 36 inches apart, the subject leaned over to touch one hand on the other and then returned the hand to the starting position. Tape marked the starting position for each hand and a folded towel was placed under the knees for comfort of female subjects only.	CRITERIA:<br>2 – 20 repetitions<br>1 – less than 20<br>0 – painful	Likert 0 to 2
	Movement System Screening Tool	Two lines of tape are placed 36 inches apart on the floor. Participant starts the test in a standard push-up position, with one hand on each tapeline. Participant is allowed a practice trial to ensure proper form, defined as: feet shoulder width apart; shoulders, hips, knees and ankles aligned in the coronal plane; and each hand must touch the opposite line to count as a repetition. To perform, bring one hand over to the opposite tape line, return to the starting tapeline, and then repeated the task with the opposite hand.	CRITERIA:<br>Score: Average number of touches in 15 seconds<br>0: Pain present during test.<br>1: > 2 SD from normative mean by sex<br>2: < 2, > 1 SD from normative mean by sex<br>3: < 1 SD from normative mean by sex.<br>Normative means of 21.8 ± 3.9	Likert 0 to 3 (4 points)

Y Balance Test	Y Balance Test – Upper Quarter	The posterior pipes are positioned 135 from the anterior pipe, and there is 90 between the posterior pipes. Each pipe is marked in 0.5-cm increments for measurement. The subject pushes a target (reach indicator) along the pipe, which standardizes the reach height (i.e., how far off the ground the reach hand is), and the target remains over the tape measure during performance of the test, which improves the precision in determining reach distance.<br><br>Before testing, all subjects viewed a video that provided standardized instructions, which included a demonstration by the examiner of the test position. All subjects performed the test with shoes off.<br><br>To perform the YBT-UQ, the subject assumed the starting position with testing hand on the stance platform and the thumb adducted while being aligned behind the red starting line. The starting position for the reach hand was defined by positioning the reach hand on top of the medial reach indicator placed shoulder width from the stance plate. Performance on the test consisted of the subject reaching in the 3 reach directions with the free hand while maintaining a push-up position with feet shoulder width apart. The trial was discarded and repeated if the subject (a) failed to maintain unilateral stance on the platform (e.g., touched down to the floor with the reach hand or fell off the stance platform), (b) failed to maintain reach hand contact with the reach indicator on the target area while it was in motion (e.g., shoved the reach indicator), (c) used the reach indicator for stance support (e.g., placed fingers or hand on top of the reach indicator), (d) failed to return the reach hand to the starting position under control, or (e) lifted either foot off of the floor. This process was repeated until 3 trials in each direction on each hand had been performed. Subjects were allowed to stop and remove themselves from testing at any time.	The variables of interest for the study included normalized maximum reach distances for each reach direction along with composite reach distance. The greatest successful reach for each direction for each rater was used for analysis. The maximum reach distances were divided by the subject's upper limb length to normalize each reach distance. To measure upper limb length, the subject stood in an anatomical position while the investigator identified the C7 vertebrae. After C7 was identified, the investigator instructed the subject to raise (abduct) the right limb to shoulder height (90). The examiner then measured the distance from the C7 spinous process to the most distal tip of the right middle finger (in centimeters) with a cloth tape measure. The composite reach distance was calculated by averaging the greatest trial in each of the 3 normalized reach distances for an analysis of overall performance on the test.	Normalised reach distance<br><br>Absolute Reach distance
	Movement System Screening Tool (Y Balance Test – Lower Quarter Anterior)	Participant stands with stance foot in the middle of the platform so that toes are behind the start line, hands on hips, and reach forward pushing the indicator box as far as possible in one smooth movement. After box is pushed, the distance is recorded, and box is returned to the start position for the next trial. Trials are unsuccessful if at any point the hands came off the hips, the stance heel is lifted, or a loss of balance is observed. The average of 3 trials is normalized to height.	0: Pain present during test<br>1:>2 SD from normative mean by sex<br>2: 1 SD from normative mean by sex<br>3: <1 SD from normative mean by sex<br><br>Women's scores were based on means of 0.43 ± 0.06 (left) and 0.43 ± 0.07 (right)<br>Men's scores were based on means of 0.45 ± 0.06 (left) and 0.45 ± 0.06 (right)<br>Sides scored separately.	Likert 0 to 3 (4 points possible)

Dynamic leap and balance test	Dynamic leap and balance test	Participants began at the center target of the testing matrix standing on their dominant limb with the nondominant limb foot next to the stance leg medial malleolus. The test was initiated by a verbal command of "Go" from the lead investigator. The participant then leaped from their dominant limb to a predetermined target landing on their non-dominant limb. The operational definition for a leap was "an acceleration or taking off from one limb and landing on the other limb". Once on the peripheral target, the participant immediately leaped back to the central target, landing on their dominant limb and trying to attain and then maintain balance for two seconds.<br>Once the investigator noted a restoration of balance for two seconds an audible command of "Go" was given to indicate the participant could leap to the next peripheral target. Participants continued this pattern of leaping and balancing for a total of 20 leaps (five directions and two distances in each direction). If the participant missed the target upon landing, they were instructed to reposition on the target as quickly as possible. All participants began with the anterior direction and moved in a clockwise (left leg dominant) or counterclockwise (right leg dominant) manner through all of the matrix directions, finishing after leaping from the posterior direction. In each direction, the participant leaped to the short target before the long target.<br><br>Verbal instructions were given and 1 demonstration. 3 practice trials before 3 timed trials.	Attainment of balance was assessed using criteria similar to the modified Balance Error Scoring System (BESS) criteria (1) touching down with opposite foot, (2) excessive hip abduction, (3) out of testing position for more than two seconds and/or (4) step, stumble or fall)<br>Total time (seconds), to complete this task, was measured using a stop watch by the same investigator for each trial.	Average time across the three trials
Spinal Extension Clearing Exam	Functional Movement Screen	Assume the "upward dog" yoga position.	Pain = positive test = 0 on trunk stability push up test	Pass/fail
Rotary Stability Test	Functional Movement Screen<br><br><br>Modified Functional Movement Screen (1)	The individual assumes the starting position in quadruped, their shoulders and hips at 90-degree angles, relative to the torso, with the 2 x 6 board between their hands and knees. The knees are positioned at 90 degrees and the ankles should be dorsi-flexed. The individual then flexes the shoulder and extends the same side hip and knee. The leg and hand are only raised enough to clear the floor by approximately 6 inches. The same shoulder is then extended and the knee flexed enough for the elbow and knee to touch. This is performed bilaterally, for up to three attempts each side. If the individual cannot complete this manoeuvre (score a "3"), they are then instructed perform a diagonal pattern using the opposite shoulder and hip in the same manner as described for the previous test	0 = pain or pain during the spinal flexion clearing test<br>1 – no pain and completing diagonal variation off balance<br>2 – completion of diagonal variation<br>3 – completion of unilateral variation	Likert 0 to 3 (4 point)

	Movement System Screening Tool	Participant assumes a quadruped position with the hips and knees at 90◦ and a 2 × 6 board between the hands and knees. With the ankles dorsiflexed, toes, knees, and thumbs touching the board, participant raises the arm and extends the ipsilateral hip and knee simultaneously. After achieving this position, participant brings the elevated elbow and knee towards the midline of the body to make contact above the board and then return to the starting position.	CRITERIA:<br>0: Pain present during test<br>1: Unable to perform the diagonal pattern<br>2: Unable to perform the ipsilateral pattern but able to perform the diagonal pattern<br>3: Able to perform the ipsilateral pattern<br>Sides scored separately.	Likert 0 to 3 (4 points)
Spinal flexion clearing test<br><br>Spinal flexion/extension test	Functional Movement Screen<br><br>Movement System Screening Test	Assume the "child's pose" from yoga<br><br>For the trunk flexion test, participant assumes a quadruped position and are instructed to rock his/her hips toward his/her heels, lower the chest to his/her knees, and reach his/her hands in front of the body as far as possible. For the trunk extension test, participant lies prone on an exercise mat with the hands directly under the shoulders and the feet together. From this position, participant presses his/her chest off the mat as much as possible by straightening the elbows and without any lower body movement. Raters observe for limited or excessive motion in the shoulder (flexion test only), thoracic and lumbar spines, and hips.	Positive if pain<br><br>0: Pain present during either test<br>1: Limited or excessive motion observed on both tests<br>2: Limited or excessive motion observed on only one test<br>3: No limited or excessive motion was observed on either test	Pass/Fail<br><br>Likert 0 to 3 (4 point)
Lateral step down test	Lateral step down test	The subject was asked to stand in single limb support with the hands on the waist, the knee straight and the foot positioned close to the edge of a 20 cm high step. The contralateral leg was positioned over the floor adjacent to the step and was maintained with the knee in extension. The subject then bent the tested knee until the contralateral leg gently contacted the floor and then re-extended the knee to the start position. This maneuver was repeated for 5 repetitions.	The examiner faced the subject and scored the test based on 5 criteria:<br>1) Arm strategy. If subject used an arm strategy in an attempt to recover balance, 1 point was added;<br>2) Trunk movement. If the trunk leaned to any side, 1 point was added<br>3) Pelvis plane. If pelvis rotated or elevated one side compared with the other, 1 point was added<br>4) Knee position. If the knee deviated medially and the tibial tuberosity crossed an imaginary vertical line over the 2nd toe, add 1 point, or, if the knee deviated medially and the tibial tuberosity crossed an imaginary vertical line over the medial border of the foot, add 2 points,<br>5) Maintain steady unilateral stance. If the subject stepped down on the non-tested side, or if the subject tested limb became unsteady (i.e. wavered from side to side on the tested side), add 1 point.<br><br>Total score of 0 or 1 was classified as good quality of movement, total score of 2 or 3 was classified as medium quality, and total score of 4 or above was classified as poor quality of movement.	Likert 1 to 5
Sprint Run	Fundamental Movement Skills Test	Participants are asked to run a 30 m track as fast as they can. Assessment can either occur live or via video recorded with a camera placed side on and far enough away the operator can move the camera as the participant runs from one end to another	Criteria:<br>1) Eyes focused throughout the run<br>2) Knees bend at right angles during the recovery phase<br>3) Arms bend at elbows and move in opposition to legs<br>4) Contact ground with front part of foot<br>5) Body leans slightly forward	Pass/fail on each criteria, score out of 5

Vertical Jump	Fundamental Movement Skills test	Jump as high as possible from a standing position. Must take off and land on the same mark	Criteria:<br>1) Eyes focused forwards or upwards throughout the jump<br>2) Crouch with knees bent and arms behind body<br>3) Forceful upwards thrust of arms as legs straighten to take off<br>4) Contact ground with front part of feet and bend knees to absorb force of landing<br>5) Balanced landing with no more than one step in any direction	Pass/fail on each criteria, score out of 5
Overarm Throw	Fundamental Movement Skills test	Mark a 2 metre square with cones. Place a target marker 10-20 m away. Participant is to throw the beanbag hard overhand towards the target. 6 throws can be undertaken.	Criteria:<br>1) Eyes are focused on the target throughout the throw<br>2) Stand side-on to target<br>3) Throwing arm nearly straightened behind the body<br>4) Step toward the target with foot opposite throwing arm during the throw<br>5) Marked sequential hip to shoulder rotation during the throw<br>6) Throwing arm follows through down and across the body	Pass/fail on each criteria, score out of 6
Catch	Fundamental Movement skills test	Objective is to catch a tennis ball thrown underarm from a specified distance (5 metres – 5 to 7 y, 10 m – 8 to 10 y, 15 m – 11 to 12 y)<br>Measure a 2 metre square, place at least 6 tennis balls next to the position where the ball is being thrown. Throw the ball at 2-3 m above the ground. Instruct the participant to catch the ball not leaving the 2 metre square and place next to them.	Criteria:<br>1) Eyes are focused on the ball throughout the catch<br>2) Preparatory position with elbows bent and hands in front of the body<br>3) Hands move to meet the ball<br>4) Hands and fingers positioned correctly to catch the ball<br>5) Catch and control the ball with hands only<br>6) Elbows bend to absorb force of the ball	Pass/fail on each criteria, score out of 6
Kick	Fundamental Movement Skills Test	Place a mark from where the ball is to be kicked on a non-slip surface. Draw a line 3 metres back from the mark as the starting point. Place a ball on the marked spot. Ask the participant to start at the starting point and kick the ball as hard as they can at the target (10-20 m away)	Criteria:<br>1) Eyes are focused on the ball throughout the kick<br>2) Step forward with non-kicking foot placed near the ball<br>3) Bend knee of kicking leg during the backswing for the kick<br>4) Hip extension and knee flexion of at least 90 during preliminary kicking movement<br>5) Contact the ball with the top of the foot<br>6) Forward and sideward swing of arm opposite kicking leg<br>7) Kicking leg follows through towards the target after ball contact	Pass/fail on each criteria, score out of 7
Forehand strike	Fundamental Movement Skills Test	Measure a 2 x 2 metre cone square, mark back line as starting position. Mark spot 5-10 metres from which to throw the ball to be struck. Place racquet next to hit zone and at least 6 balls next to throw mark.<br><br>Demonstrate requirements of forehand strike.<br>Instruct participant to stand in starting position and strike the ball so that it travels on the full past the thrower. Return to starting position after each strike	Criteria:<br>1) Eyes are focused on the ball throughout the strike<br>2) Stand side-on to the target with bat held in one hand<br>3) Striking hand nearly straightened behind shoulder at end of backswing<br>4) Step toward target with foot opposite striking arm during the strike<br>5) Marked sequential hip to shoulder rotation during the strike<br>6) Ball contact made opposite front foot with straight arm<br>7) Follow through towards the target then around the body	Pass/fail on each criteria, score out of 7

Drop Jump	Landing Error Scoring System	The jump-landing task incorporated vertical and horizontal movements as participants jumped from a 30-cm high box to a distance of 50% of their height away from the box, down to a force platform, and immediately rebounded for a maximal vertical jump on landing (Figure 1). During task instruction, emphasis was placed on subjects jumping as high as they could once they landed from the box. Subjects were not provided any feedback or coaching on their landing technique unless they were performing the task incorrectly. After task instruction, the subject was given as many practice trials as needed (typically 2) to perform the task successfully. A successful jump was characterized by (1) jumping off of both feet from the box; (2) jumping forward, but not vertically, to reach the force plate below; (3) landing with the entire foot of the dominant lower extremity on the force plate; (4) landing with the entire foot of the nondominant lower extremity off the force plate; and (5) completing the task in a fluid motion.<br><br>2D video capture in the frontal/sagittal plane occurs at 136 inches from the front and side of the landing zone respectively	Criteria:<br>1) Knee flexion angle at initial contact<br>a. At the time point of initial contact, if the knee of the test leg is flexed more than 30 degrees, score YES. If the knee is not flexed more than 30 degrees, score NO<br>2) Hip flexion angle at initial contact<br>a. At the time point of initial contact, if the thigh of the test leg is in line with the trunk then the hips are not flexed and score NO. If the thigh of the test leg is flexed on the trunk, score YES.<br>3) Trunk flexion angle at initial contact<br>a. At the time point of initial contact, if the trunk is vertical or extended on the hips, score NO. If the trunk is flexed on the hips, score YES.<br>4) Ankle plantarflexion angle at initial contact<br>a. If the foot of the test leg lands toe to heel, score YES. If the foot of the test leg lands heel to toe or with a flat foot, score NO.<br>5) Knee valgus angle at initial contact<br>a. At the time point of initial contact, draw a line straight down from the center of the patella. If the line goes through the midfoot, score NO. If the line is medial to the midfoot, score YES.<br>6) Lateral trunk flexion angle at initial contact<br>a. At the time point of initial contact, if the midline of the trunk is flexed to the left or the right side of the body, score YES. If the trunk is not flexed to the left or right side of the body, score NO.<br>7) Stance width – Wide<br>a. Once the entire foot is in contact with the ground, draw a line down from the tip of the shoulders. If the line on the side of the test leg is inside the foot of the test leg then greater than shoulder width (wide), score YES. If the test foot is internally or externally rotated, grade the stance width based on heel placement.<br>8) Stance width – Narrow<br>a. Once the entire foot is in contact with the ground, draw a line down from the tip of the shoulders. If the line on the side of the test leg is outside of the foot then score less than shoulder width (narrow), score YES. If the test foot is internally or externally rotated, grade the stance width based on heel placement.<br>9) Foot position - Toe In<br>a. If the foot of the test leg is internally more than 30 degrees between the time period of initial contact and max knee flexion, then score YES. If the foot is not internally rotated more than 30 degrees	Scoring:<br>1) Yes = 0, No = 1<br>2) Yes = 0, No = 1<br>3) Yes = 0, No = 1<br>4) Yes = 0, No = 1<br>5) Yes = 1, No = 0<br>6) Yes = 1, No = 0<br>7) Yes = 1, No = 0<br>8) Yes = 1, No = 0<br>9) Yes = 1, No = 0<br>10) Yes = 1, No = 0<br>11) Yes = 0, No = 1<br>12) Yes = 0, No = 1<br>13) Yes = 0, No = 1<br>14) Yes = 0, No = 1<br>15) Yes = 1, No = 0<br>16) Soft = 0, Average = 1, Stiff = 2<br>17) Excellent = 0, Average = 1, Poor = 2

			between the time period of initial contact to max knee flexion, score NO.<br>10) Foot position - Toe Out<br>a. If the foot of the test leg is externally rotated more than 30 degrees between the time period of initial contact and max knee flexion, then score YES. If the foot is not externally rotated more than 30 degrees between the time period of initial contact to max knee flexion, score NO.<br>11) Symmetric initial foot contact<br>a. If one foot lands before the other or if one foot lands heel to toe and the other lands toe to heel, score NO. If the feet land symmetrically, score YES.<br>12) Knee flexion displacement<br>a. If the knee of the test leg flexes more than 45 degrees from initial contact to max knee flexion, score YES. If the knee of the test leg does not flex more than 45 degrees, score NO.<br>13) Hip flexion at max knee flexion<br>a. If the thigh of the test leg flexes more on the trunk from initial contact to max knee flexion angle, score YES.<br>14) Trunk flexion at max knee flexion<br>a. If the trunk flexes more from the point of initial contact to max knee flexion, score YES. If the trunk does not flex more, score NO.<br>15) Knee valgus displacement<br>a. At the point of max knee valgus on the test leg, draw a line straight down from the center of the patella. If the line runs through the great toe or is medial to the great toe, score YES. If the line is lateral to the great toe, score NO.<br>16) Joint displacement<br>a. Watch the sagittal plane motion at the hips and knees from initial contact to max knee flexion angle. If the subject goes through large displacement of the trunk, hips, and knees then score SOFT. If the subject goes through some trunk, hip, and knee displacement but not a large amount, then AVERAGE. If the subject goes through very little, if any trunk, hip, and knee displacement, then STIFF.<br>17) Overall impression<br>a. Score EXCELLENT if the subject displays a soft landing and no frontal plane motion at the knee, Score POOR if the subject displays a stiff landing and large frontal plane motion at the knee. All other landings, score AVERAGE.	

Small knee bend<br><br><br><br><br><br><br>Single Leg Small Knee bend	Lower Extremity Functional Tests<br><br><br><br><br><br><br>Lower Extremity Functional Tests	Starting from a standing position, individuals performed a partial squat (hip and knee flexion) with the trunk maintained in an upright position. Individuals were instructed to continue the SKB until they reached maximum dorsiflexion without lifting their heels and then return to upright standing<br><br>Standing on the dominant leg only, with the contralateral hip in neutral and contralateral knee flexed to approximately 80°, individuals performed a SKB as described above.	Criteria:<br>Trunk – moves out of neutral in frontal or transverse plane<br><br>Pelvis 1 – moves out of neutral in the frontal or transverse plane<br><br>Pelvis 2 – moves away from the midline<br><br>Knee – Patella moves out of line with 2nd toe<br><br>Foot – Moves into excessive pronation<br><br>Oscillation – Observable oscillation (movement to and from neutral)<br><br>Overall movement quality	Scoring: Likert 0 to 3 with different criteria<br>Trunk – No = 0, Yes (minor) = 1, Yes (moderate) = 2, Yes (marked) = 3<br>Pelvis 1 – No = 0, Yes (minor) = 1, Yes (moderate) = 2, Yes (marked) = 3<br>Pelvis 2– No = 0, Yes (minor) = 1, Yes (moderate) = 2, Yes (marked) = 3<br>Knee – No = 0, Yes (minor) = 1, Yes (moderate) = 2, Yes (marked) = 3<br>Foot – No = 0, Yes (minor) = 1, Yes (moderate) = 2, Yes (marked) = 3<br>Oscillation – No = 0, Yes (minor) = 1, Yes (moderate) = 2, Yes (marked) = 3<br><br>Overall movement quality – Acceptable = 0, minor dysfunction = 1, moderate dysfunction = 2, marked dysfunction = 3
Weight bearing forward lunge<br><br><br><br><br><br><br>Weight bearing dorsi-flexion	Modified Musculoskeletal Readiness Screening Tool	Participants assumed a shoulder-width staggered stance position with two fingers touching the wall, as a balance aid only, and the tip of the forward great toe 12cm from the wall. The subject lunged forward while attempting to keep the front heel on the ground. This was repeated for the contralateral limb<br><br>Ankle dorsiflexion was evaluated through the LegMotion system (LegMotion, Check your MOtion, Albacete, Spain) (Figure 1).22 Each player started with their hands on their hips, and put the assigned foot on the middle of the longitudinal line just behind the transversal line on the platform. The alternate foot was positioned out of the platform with toes at the edge of the platform. While maintaining this position, subjects were instructed to perform a lunge in which the knee was flexed with the goal of making contact between the anterior knee and the metal stick. When subject were able to maintain heel and knee contact, the metal stick was progressed away from knee. The distance achieved was recorded in centimeters. Three trials were allowed with each ankle (i.e., left and right) with 10 seconds of passive recovery between trials. The third value in each ankle was selected for subsequent analysis of weight-bearing dorsiflexion (WB-DF).	CRITERIA:<br>2 – patellae contacted the wall for both legs<br>1 – patellar contacted the wall for one leg<br>0 – painful or unable to touch wall	Likert 0 to 2<br><br><br><br><br><br><br>Distance from wall
Unilateral Wall Sit Hold	Musculoskeletal Readiness Screening Tool	The unilateral wall sit hold required the subject to stand with body weight evenly distributed, feet shoulder width apart, and shoes on. The back was pressed against the wall with the hips and knees flexed to create an angle between the wall and thigh at 45 degrees. The arms	CRITERIA:<br>2 – maintaining position 30 seconds bilaterally<br>1 – holding less than 30 seconds on either leg<br>0 – if test was painful	Likert 0 to 2

		hung vertically and then the subject lifted one foot such that it was 1-2 inches off the floor. The investigator started the stopwatch and then stopped at 30 seconds or when the athlete could not sustain the test position. One minute of rest was followed by testing of the contralateral limb.		
Feagin Hop (SL Vertical Hop)	Musculoskeletal Readiness Screening Tool	The Feagin hop test involved the subject standing directly on a line with the non-test lower extremity held in slight knee flexion with the shoes on. The subject performed a maximum effort vertical hop. This was performed twice on each leg.	CRITERIA:<br>2 – landing in same position with soft landing and no frontal plane deviation bilaterally<br>1 – did not meet criteria above<br>0 - painful	Likert 0 to 2
Object Manipulation Assessments	Movement Competency Assessment for Children	Two-handed catch<br>Throw (Right and Left)<br>Kick (Right and left)<br>Strike (Forehand and backhand with shoulder and hip rotation)	Not specified	Not specified
Locomotor	Movement Competency Assessment for Children	Run<br>Cut/Dodge (body position/knee valgus)<br>Deceleration (body control)<br>Vertical jump and land<br>Horizontal jump and land<br>Diagonal hop (knee valgus)<br>Skip	Not specified	Not specified
Stability	Movement Competency Assessment for Children	Beam balance (with 360 turns)<br>Single-leg balance<br>Core stability (bear crawl and crab walk)<br>Single-leg vestibular functioning (tennis ball throw)<br>Vestibular functioning test on a line (turning head)	Not specified	Not specified
Standing Posture Assessment	Movement Competency Screen	Please stand facing the camera with your hands by your side. (Hold athlete for three seconds.) Please turn to the side with your hands to the side. (Hold athlete for three seconds.)	CRITERIA:<br>Head – Held in neutral position<br>Shoulders – Held down away from ears. Slight flexion of thoracic spine OK.<br>Lumbar – Held in neutral curve position.<br>Hips – Appear to be horizontally aligned.<br>Knees – Knee caps pointing forward.<br>Ankles – NR<br>Feet – Pointing straight.<br>Balance – Evenly distributed<br>Depth – NR	If no then recorded as a point – more points = poorer function
Bend-and-pull	Movement Competency Screen	Start with your arms stretched overhead. Bend forward allowing your arms to drop under your trunk. Pull your hands into your body as if you were holding onto a bar and performing a barbell rowing exercise. Return to the start position with your arms stretched overhead.	CRITERIA:<br>Head – Held in neutral position<br>Shoulders – Held down and away from ears. Scapulae movement balanced and rhythmic. During arm flexion scapulae are retracted and are not excessively abducted during arm extension.<br>Lumbar – Held in neutral curve position throughout trunk flexion.<br>Hips – Facilitate trunk flexion.<br>Knees – Extended.<br>Ankles – NR<br>Feet – Pointing straight.<br>Balance – Maintained	If no then recorded as a point – more points = poorer function

			Depth – 75-90 degrees of trunk flexion achieved.	
Double leg lowering test	Movement System Screening Tool	Participant lies on his/her back with legs straight and hips flexed to 90◦. From this position, slowly lower the legs towards the floor without changing the pressure in the blood pressure cuff under their lumbar spine. Once the pressure changes 10 mm Hg, the angle of the legs relative to the horizontal is recorded.	0: Pain present during test<br>1: > 2 SD from normative mean by sex<br>2: 1 SD from normative mean by sex<br>3: < 1 SD from normative mean by sex<br><br>Women's scores based on means of 52.0◦ ± 5.0◦<br>Men's scores based on means of 46.0◦ ± 3.0◦	Likert 0 to 3 (4 points)
Scapular dyskinesis	Movement System Screening Tool	Test uses 5 repetitions of bilateral, active shoulder flexion and abduction. Each motion is demonstrated, and participants can perform a few practice trials. Participant performs the movements with a 2-pound (lb) (< 150 lbs) or 4-lb (> 150 lbs) weight in each in hand. Participant begins standing in a neutral position, arms at the side of the body, elbows straight, and thumbs pointing up. Raters stand 2 to 3 m behind the participant to observe scapular motion	0: Pain present during test<br>1: Obvious winging or dysrhythmia observed on 3/5 trials in either flexion or abduction<br>2: Subtle winging or dysrhythmia observed on 3/5 trials in either flexion or abduction<br>3: No winging or dysrhythmia was observed<br>Sides scored separately	Likert 0 to 3 (4 points)
Glenohumeral internal rotation deficit	Movement System Screening Tool	The measurement includes passive range of motion of shoulder internal and external rotation. Participant is supine. The start position (0◦) for both internal and external rotation measures is as follows: shoulder in 90◦ of abduction, elbow flexed to 90◦, forearm in neutral with the participant's hand/fingers pointing up towards the ceiling. From this position, the tester moves the glenohumeral joint into external or internal rotation.	0: Pain present during test<br>1: If GIRD is present (non-dominant - dominant IR difference > 14◦ AND non-dominant - dominant total arc difference > 10◦)<br>2: > 10◦ difference in total arc motion between sides, but IR difference < 14◦<br>3: < 10◦ difference in total arc motion between sides	Likert 0 to 3 (4 points)
Unilateral hip bridge endurance	Movement System Screening Tool	Participant lies supine with the arms across the chest, knees in 90◦ of flexion, and feet flat on the table (Figure 3c). Participant performs a single-leg hip bridge and holds this position as long as possible. The test is terminated when he/she is no longer able to maintain a neutral pelvic position as noted by 10◦ change in transverse or sagittal plane alignment. Three trials are performed on each side. Sides are scored individually	0: Pain present during test.<br>1: > 1 SD from normative mean by sex<br>2: ½ SD from normative mean by sex<br>3: < ½ SD from normative mean by sex<br><br>Scores were based off the following means and standard deviations:<br>females = 33.45 ± 29.70 s<br>males = 26.01 ± 18.44 s	Likert 0 to 3 (4 points)
Clinical Core Control tests (3 Cs)	Movement System Screening Tool	Participant sits atop a 75 cm Swiss ball with arms crossed and feet on ground, so knees, hips, and ankles are at 90◦. Lift the feet from the ground, keeping the heels in contact with the ball, and attempt to maintain the testing position without allowing the feet to touch the ground or ball to touch the wall 6 inches behind. Test is terminated when feet touch ground or ball touches the wall.	0: Pain present during test.<br>1: > 1 SD from normative mean by sex<br>2: < 1, > ½ SD from normative mean by sex<br>3: < ½ SD from normative mean by sex<br><br>Scores based off means and standard deviations:<br>Females = 50.12 ± 45.01 s<br>Males = 57.54 ± 59.92 s	Likert 0 to 3 (4 points)

Suspended Row	Resistance Skills Training Battery	Provide a demonstration of the movement. Instruct the participant to perform 4 repetitions starting with their upper body at a 45-60 degree angle. Repeat a second trial.	CRITERIA:<br>1. Straight line through head and back<br>2. . Body is pulled upwards to touch handles or bar at chest height<br>3. Arms are fully extended in the bottom position<br>4. No bending at the hips	Likert 1 to 3 for overall movement<br>3 = high performance (all performed correctly)<br>2 = moderate (most performed correctly)<br>1 = low (few performed correctly)<br><br>Best repetition method used
Standing Overhead Press	Resistance Skills Training Battery	Provide a demonstration of the movement. Instruct the participant to perform 4 repetitions. Repeat a second trial.	CRITERIA:<br>1. Bar is gripped slightly wider than shoulders<br>2. Back is kept straight and stable throughout movement<br>3. Bar starts at chest height and is pressed upward until arms are fully extended<br>4. Bar remains parallel to the ground throughout the movement<br>5. Bar is overhead at the top of the lift	Likert 1 to 3 for overall movement<br>3 = high performance (all performed correctly)<br>2 = moderate (most performed correctly)<br>1 = low (few performed correctly)<br><br>Best repetition method used
Active Cervical Flexion	Selective Functional Movement Assessment	Stand in a tall position with feet together and toes pointing forward. Please bring your chin down to your chest with your body upright	Can't touch sternum	Either 1 point for each criteria fulfilled OR<br><br>Categorised as:<br>Functionally non-painful<br>Functional-Painful<br>Dysfunctional-Non Painful<br>Dysfunctional-Painful
Active Cervical Extension	Selective Functional Movement Assessment	Stand in a tall position with feet together and toes pointing forward. Please look up to the ceiling.	Greater than 10 degrees of parallel	Either 1 point for each criteria fulfilled OR<br><br>Categorised as:<br>Functionally non-painful<br>Functional-Painful<br>Dysfunctional-Non Painful<br>Dysfunctional-Painful
Cervical Rotation Side-Bend	Selective Functional Movement Assessment	Stand in a tall position with feet together and toes pointing forward. Please turn your head as far as you can to the right/left and then bend down and touch your chin towards your collarbone.	Can't touch chin to mid-clavicle – SCORE BOTH SIDES	Either 1 point for each criteria fulfilled OR<br><br>Categorised as:<br>Functionally non-painful<br>Functional-Painful<br>Dysfunctional-Non Painful<br>Dysfunctional-Painful
Upper Extremity Pattern 1: Medial Rotation Extension	Selective Functional Movement Assessment	Stand in a tall position with feet together and toes pointing forward. Take your Right/Left arm and reach behind your back and aiming to touch the bottom of your opposite shoulder blade.	Can't touch inferior angle of contralateral scapula<br>Can't touch spine of the contralateral scapula<br><br>SCORE BOTH SIDES	Either 1 point for each criteria fulfilled OR<br><br>Categorised as:<br>Functionally non-painful<br>Functional-Painful<br>Dysfunctional-Non Painful<br>Dysfunctional-Painful

Upper Extremity Pattern 2: Lateral Rotation Extension	Selective Functional Movement Assessment	Stand in a tall position with feet together and toes pointing forward. Take your Right/Left arm and reach up and behind your head towards the top of your opposite shoulder blade.	Can't touch inferior angle of contralateral scapula<br>Can't touch spine of the contralateral scapula<br><br>SCORE BOTH SIDES	Either 1 point for each criteria fulfilled OR<br><br>Categorised as:<br>Functionally non-painful<br>Functional-Painful<br>Dysfunctional-Non Painful<br>Dysfunctional-Painful
Multi-segmental Flexion	Selective Functional Movement Assessment	Stand in a tall position with feet together and toes pointing forward. Please bend down and try to touch your toes.	Can't touch toes and return to standing position<br>< 70 degrees sacral angle<br>No posterior weight shift (T-L Junction over foot)<br>Non-Uniform Spinal curves	Either 1 point for each criteria fulfilled OR<br><br>Categorised as:<br>Functionally non-painful<br>Functional-Painful<br>Dysfunctional-Non Painful<br>Dysfunctional-Painful
Multi-segmental Extension	Selective Functional Movement Assessment	Stand in a tall position with feet together and toes pointing forward. Please keep your hands above your head and reach back as far as you can.	ASIS doesn't clear the toes<br>Can't maintain normal (> 170 deg) shoulder flexion<br>Spine of scapula doesn't clear the heels<br>Non-uniform spinal curves	Either 1 point for each criteria fulfilled OR<br><br>Categorised as:<br>Functionally non-painful<br>Functional-Painful<br>Dysfunctional-Non Painful<br>Dysfunctional-Painful
Multi-segmental Rotation	Selective Functional Movement Assessment	Stand in a tall position with feet together and toes pointing forward. Please place your hands by your side and rotate your entire body Right/Left trying to look behind you while keeping your feet still.	Pelvis rotation < 50 degrees<br>Trunk/shoulder < 50 degrees more than pelvis<br>Spinal/pelvic deviation<br>Excessive knee flexion<br><br>SCORE BOTH SIDES	Either 1 point for each criteria fulfilled OR<br><br>Categorised as:<br>Functionally non-painful<br>Functional-Painful<br>Dysfunctional-Non Painful<br>Dysfunctional-Painful
Single Leg Stance (eyes open, then closed)	Selective Functional Movement Assessment	Stand in a tall position with feet together and toes pointing forward.<br>1. Lift your Right/Left leg so that your hip and knee make 90 degree angles. Please hold this for 10 seconds.<br>2. Now lift your leg to the same 90 degree position and then close your eyes. Hold this position for 10 seconds.	Eyes open standing < 10 seconds<br>Eyes closed standing < 10 seconds<br>Loss of height	Either 1 point for each criteria fulfilled OR<br><br>Categorised as:<br>Functionally non-painful<br>Functional-Painful<br>Dysfunctional-Non Painful<br>Dysfunctional-Painful
